# Co-Pyrolysis of Waste Tires and Beech Sawdust: Comprehensive Analysis of Thermal Behavior, Synergistic Effect, and Interaction Mechanisms

**DOI:** 10.3390/ma19081495

**Published:** 2026-04-08

**Authors:** Guangyao Zheng, Chengyang Cao, Qiming Zhang, Pei Jia, Lu Dong, Hongyun Hu

**Affiliations:** 1 School of Resource & Safety Engineering, Wuhan Institute of Technology, Wuhan 430074, China; zgy837080481@163.com (G.Z.); 04001074@wit.edu.cn (P.J.); 2 State Key Laboratory of Low Carbon Catalysis and Carbon Dioxide Utilization, School of Petroleum Engineering, Yangtze University, Wuhan 430100, China; ludong@hust.edu.cn; 3 State Key Laboratory of Coal Combustion, Huazhong University of Science and Technology, Wuhan 430074, China; hongyunhu@hust.edu.cn

**Keywords:** co-pyrolysis, waste tires, beech sawdust, synergistic effect, tar

## Abstract

**Highlights:**

**What are the main findings?**

- Beech sawdust lowers the decomposition temperature of waste tire blends.
- Co-pyrolysis increases tar yield to 64.45 wt.% while reducing char residue.
- Aromatic hydrocarbon production is synergistically enhanced by up to 54.8%.
- Hydrogen radicals from beech sawdust promote stable alkylbenzene formation.

**What are the implications of the main findings?**

- Co-pyrolysis serves as an effective method for high-value waste tire utilization.
- Beech sawdust improves the quality and stability of tar.
- The study offers theoretical insights for upgrading waste tire recycling technologies.

**Abstract:**

Against the backdrop of the global search for alternatives to fossil fuels, waste tires have attracted attention as a significant resource due to their enormous production volume and considerable energy potential. However, the application of tar derived from waste tires alone is limited by its poor stability and other deficiencies. This study systematically investigates the co-pyrolysis behavior and synergistic mechanisms of waste tires and beech sawdust at various blending ratios. Thermogravimetric analysis indicates that the addition of beech sawdust reduces the decomposition temperature of the blend and induces a synergistic effect that promotes waste tire pyrolysis within the temperature range of 384–440 °C. Pyrolysis experiments results show that tar yield of the blends reached 64.45 wt.%, while the char yield decreased from 40.67 wt.% to 24.83 wt.%. Also, the presence of beech sawdust synergistically enhanced the formation of aromatic hydrocarbons in the tar of waste tires, with the total yield of aromatics increasing synergistically by up to 54.8%. Specifically, the yields of stable alkylbenzenes such as toluene and xylene were consistently promoted, whereas the yields of unsaturated aromatics such as allylbenzene and 2,4-dimethylstyrene were enhanced at low beech sawdust ratios but suppressed at higher ratios. Based on these findings, the interaction mechanisms underlying the co-pyrolysis process were elucidated, providing theoretical guidance for the high-value utilization of waste tires.

## 1. Introduction

Under the background of rapid transformation and upgrading of the global energy structure, the demand for seeking green and sustainable alternatives to fossil fuels has emerged as a key driving force during the current development phase. Among various solid wastes, waste tires have been regarded as one of the most important alternative fuel feedstocks, whose annual production is approximately 1.5 billion and continuously increases with the proceeding increase in automobile ownership [1,2].

Waste tires are primarily composed of natural rubber (NR), butadiene rubber (BR), and styrene-butadiene rubber (SBR), with a high carbon content of up to 82.3%, making it a resource with considerable recovery value [3,4]. Thermochemical conversion, particularly pyrolysis, is regarded as the promising technology for converting waste tires into valuable tar fuels and chemicals [5,6]. During pyrolysis, waste tires are decomposed into three primary products: char, tar and gas [7]. Pyrolytic char can be utilized to produce adsorbents such as activated carbon [8]; gas can provide sufficient energy for the pyrolysis process; and pyrolytic tar can be considered a promising substitute for conventional tar fuels due to its high calorific value (44.20–45.09 MJ/kg) and physicochemical properties similar to diesel, including density and viscosity [9,10,11,12]. Pyrolysis not only enables the complete treatment of waste rubber but also produces products with substantial application potential [13]. However, the direct application of tire tar remains limited by its intrinsic quality deficiencies, such as high sulfur content and storage instability caused by the presence of unsaturated compounds. Previous studies have demonstrated that co-pyrolysis with biomass can effectively improve the tar quality. Farooq et al. report that co-pyrolysis of waste tires with wheat straw reduced the content of aldehydes and ketones in the tar, thereby enhancing its stability [14]. Similarly, Alvarez et al. observed that adding pine sawdust during co-pyrolysis produced a stable, single-phase tar with higher carbon content and lower oxygen and water contents [15]. These studies indicate that co-pyrolysis with biomass is an effective approach to upgrading the quality of tar.

Due to the distinct physicochemical properties of rubber and biomass, numerous researchers have attempted to optimize product quality through co-pyrolysis. Shah et al. found that co-pyrolysis of cotton stalks and waste tires at a 2:3 ratio increased the tar yield from 38 wt.% to 48 wt.% [16]. Wang et al. investigated the co-pyrolysis of waste tires and corn stalks, reporting that the addition of corn stalks suppressed the formation of polycyclic aromatic hydrocarbons while increasing the yields of hydrocarbons and alcohols [17]. Martinez et al. observed a 20% increase in aromatic hydrocarbon content and a 15% reduction in acidic compounds in the tar during the co-pyrolysis of pinewood and tires [18]. Similarly, Khan et al. report that co-pyrolysis of rice straw and tires effectively reduced oxygenated compounds while increasing the proportion of aromatic and aliphatic hydrocarbons in the tar [19]. Furthermore, existing literature has also demonstrated that co-pyrolysis with biomass can effectively facilitate the desulfurization of tire-derived oil [20]. Although these studies have demonstrated the significant potential of co-pyrolysis for improving pyrolysis product quality, most have focused on process parameter optimization and quantitative analysis of synergistic effects, while in-depth investigations of the underlying synergistic mechanisms remain limited.

To address this gap, this study systematically investigates the co-pyrolysis behavior of waste tires and beech sawdust at different blending ratios. Thermogravimetric analysis is first employed to reveal the overall thermal decomposition behavior and weight loss characteristics of the blends, providing a preliminary evaluation of their synergistic effects. Subsequently, a series of fixed-bed pyrolysis experiments are conducted to quantitatively examine the influence of blending ratios on the yields of the three main products (tar, gas, and char). To gain deeper insights into the evolution of product properties, gas chromatography-mass spectrometry (GC-MS) and Fourier transform infrared spectroscopy (FTIR) are utilized to characterize the chemical composition of the products. Furthermore, generalized two-dimensional correlation spectroscopy (2D-COS) is introduced to dynamically trace the sequential evolution of chemical structures in the solid char. Finally, based on the analysis of experimental data under various blending ratios, the synergistic mechanisms of waste tires–beech sawdust co-pyrolysis are elucidated, particularly the hydrogen- and methyl-radical-driven hydrodeoxygenation and aromatization pathways, offering theoretical guidance for the high-value utilization of tire–biomass co-pyrolysis technology.

## 2. Materials and Methods

### 2.1. Materials

Waste tires and beech sawdust were collected from a recycle point in Wuhan. Both materials were crushed and sieved to obtain samples with a particle size of 1–2 mm. The samples were dried in air at 105 °C and then stored in a desiccator for later use. The prepared waste tires and beech sawdust were subsequently blended to investigate the influence of beech sawdust blending ratios on the co-pyrolysis interactions with waste tires. Pure waste tires and beech sawdust samples were denoted as waste tires and beech sawdust, respectively. Blends with mass ratios of waste tires to beech sawdust of 75:25, 50:50, and 25:75 were prepared and labeled as W75B25, W50B50, and W25B75, respectively.

The proximate analysis of waste tires was conducted according to the standard GB/T 212-2008, while that of beech sawdust was performed following GB/T 28731-2012 [21,22]. Elemental analysis was carried out using an elemental analyzer (EA, Elementar Vario Micro cube, Elementar, Langenselbold, Germany). The results are summarized in Table 1.

### 2.2. Methods

#### 2.2.1. Thermal Behavior

The pyrolysis behavior of the raw materials and their blends was characterized using thermogravimetric analysis (TGA, NETZSCH STA 449 F3, NETZSCH, Selb, Germany) [23]. The samples, including waste tires, beech sawdust, and their three blends (W75B25, W50B50, and W25B75), were heated from 30 °C to 600 °C at a heating rate of 10 °C min^−1^ under a nitrogen atmosphere with a flow rate of 100 mL min^−1^.

#### 2.2.2. Pyrolysis Experiments

**Figure 1:**
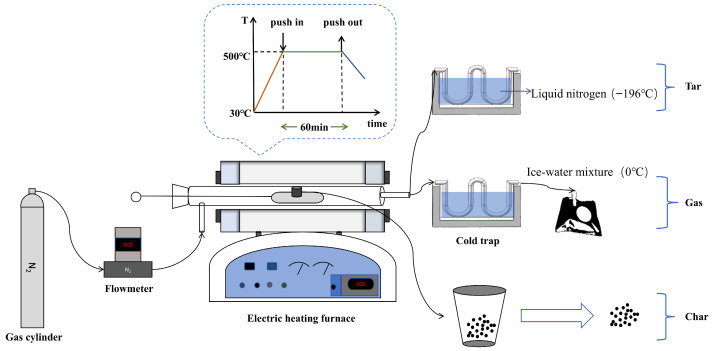
Diagram of the experimental setup.

Pyrolysis experiments of waste tires, beech sawdust, and their blends were conducted in a fixed-bed reactor. A schematic diagram of the experimental setup is shown in Figure 1. Prior to each experiment, the reactor was preheated to 500 °C and purged with nitrogen to remove air before feeding. Approximately 0.5 g of the sample was then rapidly introduced, and the pyrolysis reaction was maintained for 1 h with nitrogen at a flow rate of 600 mL min^−1^. After pyrolysis, the tar, char, and gas products were collected separately.

Char was collected from the inside of the reactor and weighed. A U-shaped tube was cooled with liquid nitrogen, the condensed tar products were recovered by rinsing the U-shaped condenser with acetone (AR, >99%, Sinopharm Chemical Reagent Co., Ltd., Shanghai, China). A U-shaped tube was cooled with an ice-water mixture, the gas products were collected in gas sampling bags, and nitrogen was used as both the carrier gas and the tracer gas for quantitative gas analysis [24].

The yields of char and gas were calculated as weight percentages of the initial feedstock using the following equation:

(1) $$Y=\frac{X_{1}}{X_{2}}\times 100\%$$

Here, *Y* represents the product yield, *X*_1_ is the mass of the product, and *X*_2_ is the initial mass of the feedstock. The tar yield was determined by difference using the following equation:

(2) $$Tar\;yield\left(\mathrm{wt}.\%\right)=100-\left(Char\;yield\left(\mathrm{wt}.\%\right)+Gas\;yield\left(\mathrm{wt}.\%\right)\right)$$

To ensure the reliability and reproducibility of the results, all fixed-bed pyrolysis experiments were performed in triplicate.

#### 2.2.3. Product Characterization Methods

The tar products were characterized using gas chromatography-mass spectrometry (GC-MS, Agilent 8860, Agilent, Santa Clara, CA, USA) equipped with a capillary column (Agilent HP-5ms, 19091S-433; 30 m × 0.25 mm × 0.25 μm). The oven temperature program was set as follows: the initial temperature was maintained at 40 °C for 5 min, followed by a ramp of 5 °C/min to 210 °C, and then 10 °C/min to 300 °C. Acetone was used as the reference solvent, and peaks with areas exceeding 1% of the total chromatogram were selected for compound identification. Although this peak area normalization method is semi-quantitative and the 1% threshold may exclude trace species, it is widely adopted as a practical tool [25,26].

The gaseous products were analyzed using a micro gas chromatograph (Agilent 7890B, Agilent, Santa Clara, CA, USA) to quantify the major pyrolysis gases, such as H_2_, CO, CO_2_, and light hydrocarbons (C_1_–C_4_). Before each experiment, the reactor was purged with argon to remove any residual gases from previous runs.

To ensure the statistical reliability of the quantitative analysis, both the GC-MS and micro-GC measurements were performed on the tar and gas products obtained from the triplicate experiments. Detailed information on the chromatographic peak area and quantification of tar and gas products is provided in the Supplementary Materials.

The FTIR spectra of char samples with different beech sawdust blending ratios (0–75%, at 25% intervals) were measured using a Fourier transform infrared spectrometer (Nicolet 6700, Thermo Fisher Scientific, Waltham, MA, USA) in the wavenumber range of 4000–500 cm^−1^ with a resolution of 2 cm^−1^.

#### 2.2.4. 2D-COS Method

To further elucidate the evolution of the chemical structure of char samples reflected by the FTIR data, generalized two-dimensional correlation spectroscopy (2D-COS) was applied in this study [27,28]. The spectral intensity $y\left(v,B\right)$ can be expressed as a function of the appropriate spectral variable (wavenumber *v*) and the external perturbation variable (blending ratio *B*). The dynamic spectrum $\tilde{y}\left(v,B\right)$ is then defined as:

(3) $$\tilde{y}\left(v,B\right)=\left\{\begin{array}{cc} y\left(v,B\right)-\bar{y}\left(v\right) & \;B_{min}\leq B\leq B_{max}\\ 0 & \;otherwise \end{array}\right.$$

where $\bar{y}\left(v\right)$ represents the reference spectrum and *B_min_* and *B_max_* denote the boundaries of the fixed blending ratio interval. Typically, the reference spectrum $\bar{y}\left(v\right)$ is taken as the average spectrum within the perturbation interval (*B_min_* → *B_max_*):

(4) $$\bar{y}\left(\nu \right)=\frac{1}{B_{max}-B_{min}}\int_{B_{min}}^{B_{max}}y\left(v,B\right)dB$$

The intensity of the 2D correlation spectrum $X\left(\nu_{1},v_{2}\right)$ is expressed as the cross-correlation function of the dynamic spectra $\tilde{y}\left(v,B\right)$, measured at different wavenumbers *ν_1_* and *ν_2_* over a fixed interval:

(5) $$X\left(\nu_{1},v_{2}\right)=\langle \tilde{y}\left(v_{1},B_{1}\right)\cdot \tilde{y}\left(v_{2},B_{2}\right)\rangle$$

Through mathematical transformation, the intensities of the synchronous $\Phi \left(\Phi_{1},\Phi_{2}\right)$ and asynchronous $\Psi \left(\Psi_{1},\Psi_{2}\right)$ 2D correlation spectra are obtained as follows:

(6) $$X\left(\nu_{1},v_{2}\right)=\Phi \left(v_{1},v_{2}\right)+\Psi \left(v_{1},v_{2}\right)=\frac{1}{\pi \left(B_{max}-B_{min}\right)}\int_{0}^{\infty }\tilde{Y_{1}}\left(\omega \right)\cdot \tilde{Y_{2}^{*}}\left(\omega \right)d\omega$$

where ω is the Fourier frequency, $\tilde{Y_{1}}\left(\omega \right)$ is the forward Fourier transform of $y\left(v_{1},B\right)$, and $\tilde{Y_{2}^{*}}\left(\omega \right)$ is the inverse Fourier transform of $y\left(v_{2},B\right)$. To determine the sequential order of functional group changes with an increasing blending ratio, the Noda rules were followed to interpret the cross-peaks and auto-peaks in the synchronous and asynchronous 2D correlation spectra.

#### 2.2.5. Synergistic Effect

The synergistic effect is used to describe the enhancement of a specific parameter or property during the co-pyrolysis of two feedstocks [29,30]. It is determined by comparing the experimental value with the theoretical value. The theoretical value is calculated based on the individual contributions of the two feedstocks and their respective mass ratios, assuming no interaction occurs between them. If the experimental value of a particular parameter or property exceeds the corresponding theoretical value, it indicates the presence of a synergistic effect, suggesting that interactions between the feedstocks enhance the parameter under investigation.

(7) $$Y_{T}=\alpha_{1}\times Y_{BS}+\alpha_{2}\times Y_{WT}$$

(8) $$∆W=Y_{E}-Y_{T}$$

(9) $$∆Y=\frac{∆W}{Y_{T}}\times 100\%$$

$\alpha_{1}$ and $\alpha_{2}$ represent the mass fractions of beech sawdust and waste tires in the blend, respectively. $Y_{BS}$ and $Y_{WT}$ denote the results obtained from the pyrolysis of beech sawdust and waste tires alone. $Y_{T}$ represents the theoretical value of the blend, $Y_{E}$ is the experimental value of the blend, ∆W indicates the difference between the experimental and theoretical values, and ∆Y reflects the extent of the synergistic effect during co-pyrolysis, expressed as a percentage (%).

## 3. Results and Discussion

### 3.1. Pyrolysis Behavior

#### 3.1.1. TG-DTG Analysis

**Figure 2:**
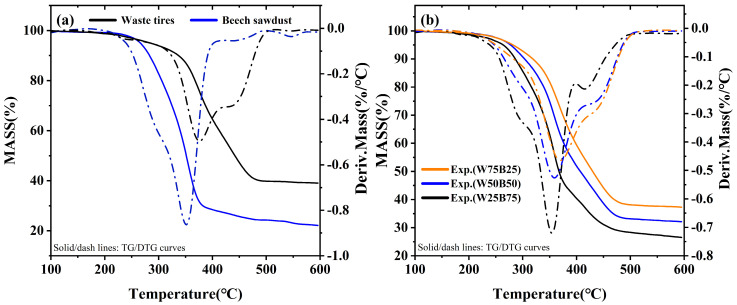
TG-DTG curves of waste tires, beech sawdust, and their blends at a heating rate of 10 °C/min under a N2 atmosphere: (**a**) waste tires and beech sawdust; (**b**) blends.

The TG-DTG curves of waste tires, beech sawdust, and their blends under a nitrogen atmosphere with a heating rate of 10 °C/min are shown in Figure 2. Figure 2a illustrates the thermal behavior of waste tires and beech sawdust during individual pyrolysis. Waste tires decomposed in the temperature range of 180–500 °C, with a total weight loss of 60.96%. Based on the DTG curve, the pyrolysis process can be divided into three stages. In the first stage (100–180 °C), waste tires exhibited a weight loss of 0.85%, corresponding to the volatilization of moisture and small molecular compounds. The second stage occurred between 180 and 500 °C, with a weight loss of 59.35% and a peak temperature at 376 °C, primarily associated with the depolymerization and secondary decomposition of macromolecular polymers in waste tires, such as natural and synthetic rubbers. The final stage, corresponding to carbonization, occurred between 500 and 600 °C, resulting in a weight loss of 0.76%.

Beech sawdust decomposed over a broader temperature range of 140–600 °C, with a total weight loss of 77.87%. Biomass pyrolysis is generally considered to result from the decomposition of hemicellulose, cellulose, and lignin. Hemicellulose decomposes between 200 and 320 °C, cellulose between 280 and 360 °C, and lignin over 140–600 °C. Two partially overlapping peaks around 300 °C can be observed in the DTG curve, likely corresponding to the decomposition of hemicellulose and cellulose [31,32].

Figure 2b shows the TG-DTG curves of the three blends (W75B25, W50B50, and W25B75). The TG curves indicate that the total weight loss gradually increases with increasing beech sawdust content: 62.30% for W75B25, 67.89% for W50B50, and 73.48% for W25B75, representing a maximum increase of 12.79%. This increase is attributed to the higher volatile content of beech sawdust compared to waste tires. The DTG curves reveal that the peak temperatures of the weight loss peaks decrease with increasing beech sawdust content, shifting from 370 °C to 360 °C and then to 355 °C, although the extent of the shift gradually diminishes.

Furthermore, as the beech sawdust proportion increases, the DTG peaks associated with the decomposition of hemicellulose and cellulose become more pronounced, indicating that biomass decomposition progressively dominates the thermal degradation of the blends. At low beech sawdust blending ratios, the pyrolysis behavior of the blend is largely influenced by waste tires. When the beech sawdust content reaches 75 wt.%, the DTG curve exhibits a peak profile similar to that of pure beech sawdust. In the low-temperature region (approximately 100–380 °C), the weight-loss behavior resembles the peaks of pure beech sawdust, suggesting that the decomposition of hemicellulose and cellulose governs the reactions within this temperature range. At higher temperatures (approximately 380–600 °C), the peak profile increasingly reflects the thermal degradation characteristics of waste tires and more thermally stable components such as lignin.

#### 3.1.2. Synergistic Effect on Co-Pyrolysis Behavior

**Figure 3:**
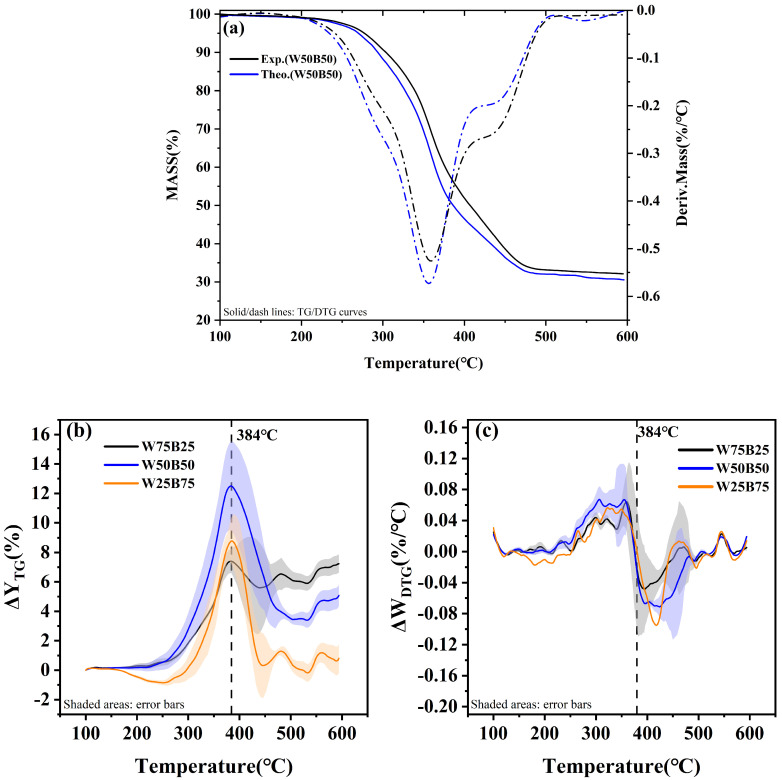
Synergistic effects of the mixtures: (**a**) W50B50; (**b**) ΔY_TG_; (**c**) ΔW_DTG_.

Figure 3a shows the TG-DTG curves of W50B50 under a nitrogen atmosphere with a heating rate of 10 °C/min, along with the corresponding theoretical curve. The blend exhibited a weight loss of 67.89%, approximately 6.93% higher than that of waste tires alone. The DTG curve displays two weight-loss peaks, with temperature ranges of 200–420 °C and 420–520 °C. Compared to the pyrolysis behavior of waste tires alone, these temperature ranges are similar; however, the addition of beech sawdust shifts the peak temperature from 376 °C to 360 °C, advancing it by 16 °C. Meanwhile, the TG curve of the blend closely follows the weighted sum of the TG curves of waste tires and beech sawdust, showing an overall inhibition of weight loss during co-pyrolysis. It suggests that the addition of beech sawdust initially suppresses the pyrolysis of waste tires. Conversely, the DTG curve indicates that before 384 °C, beech sawdust exhibits a suppressive effect on weight loss, whereas after 384 °C, a positive interaction appears, suggesting the presence of a different synergistic reaction between waste tires and beech sawdust at different temperature ranges.

The theoretical and experimental curves were used to calculate ΔY_TG_ and ΔW_DTG_, which provide a quantitative measure of the synergistic effect between waste tires and beech sawdust during co-pyrolysis. Positive values of ΔY_TG_ and ΔW_DTG_ indicate weight-loss inhibition, while negative values suggest promotion of weight loss [33]. As shown in Figure 3b, all three blends with different beech sawdust blending ratios exhibit weight-loss inhibition. Two stages can be distinguished at 384 °C. Before 384 °C, a negative synergistic effect gradually intensifies, reaching a maximum at 384 °C, with W50B50 showing the strongest inhibition. Notably, the high-beech sawdust blend W25B75 exhibits ΔY_TG_ < 0 in the 100–300 °C range, indicating a promotion of weight loss, which differs from the low-beech sawdust blends W75B25 and W50B50. From a heat-transfer perspective, it may be due to the softening of waste tires upon heating, which adheres to portions of beech sawdust and temporarily inhibits its decomposition. After 384 °C, the negative synergistic effect gradually diminishes. In the 384–440 °C range, ΔW_DTG_ fluctuates, possibly because the softened waste tires begin to decompose, allowing the previously attached beech sawdust to decompose as well [17,34]. From 440 °C to the final temperature, ΔW_DTG_ shows minor fluctuations, indicating slight interactions that have a negligible overall impact on the pyrolysis process.

### 3.2. Product Yields from Fixed-Bed Pyrolysis

**Figure 4:**
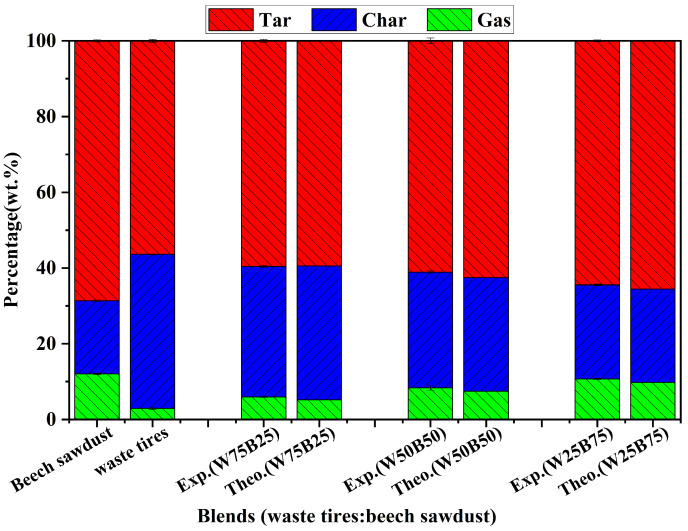
Three-phase product yields of waste tires, beech sawdust, and their blends in a fixed-bed reactor.

Figure 4 shows the distributions of the three-phase products (tar, char, and gas) obtained from the fixed-bed pyrolysis of waste tires, beech sawdust, and their blends. For the pyrolysis of waste tires alone, the yields of the three-phase products were 56.41 wt.% for tar, 40.67 wt.% for char, and 2.92 wt.% for gas. In contrast, beech sawdust produced higher tar and gas yields of 68.62 wt.% and 12.06 wt.% due to its higher volatile content [35]. It also exhibited a lower char yield of 19.32 wt.% during individual pyrolysis.

From Figure 4, it can be observed that increasing the beech sawdust blending ratio leads to an increasing trend in tar and gas yields. The tar yields of W75B25, W50B50, and W25B75 were 59.58 wt.%, 61.13 wt.%, and 64.45 wt.%, respectively, corresponding to an increase of 8.04 wt.% compared to waste tires alone. Simultaneously, the gas yield increased by 7.79 wt.%, and the char yield exhibited a significant decreasing trend, dropping by 15.84 wt.% relative to waste tires alone.

Comparing the experimental yields of the blend with the theoretical values indicates that the gas yield during co-pyrolysis slightly exceeds the theoretical value, suggesting that beech sawdust addition promotes gas formation [36]. As shown in Table 2, when the beech sawdust content exceeds 25 wt.%, the tar yield is lower than the theoretical value, while the char yield is higher, indicating that high beech sawdust content inhibits tar formation but promotes char formation. When the beech sawdust content is 25 wt.%, beech sawdust slightly promotes tar formation and enhances the overall weight loss of the blend. In all cases, beech sawdust addition consistently promotes gas generation, although the promoting effect slightly decreases with increasing beech sawdust content.

### 3.3. Product Characterization

#### 3.3.1. Tar Analysis

During individual pyrolysis, waste tires produced a tar yield of 56.41 wt.%, while beech sawdust, as a biomass with higher volatile content, yielded 68.62 wt.%, approximately 12.21 wt.% higher than waste tires alone. Experimentally, the absolute tar yield of the blends showed a clear upward trend with increasing beech sawdust blending ratios: 59.58 wt.% for W75B25, 61.13 wt.% for W50B50, and 64.45 wt.% for W25B75. This absolute increase is primarily attributed to the inherently higher tar-forming potential of beech sawdust compared to waste tires.

However, a deeper analysis comparing the experimental and theoretical yields reveals a more complex, non-monotonic synergistic effect. At a low beech sawdust blending ratio (W75B25), the experimental tar yield slightly exceeded the theoretical value, indicating a promoting effect of beech sawdust on tar formation. Conversely, at higher beech sawdust contents (W50B50 and W25B75), the experimental tar yields were slightly lower than the theoretical values by 1.12–1.39 wt.%. This negative synergistic deviation clarifies that while the absolute tar yield rises due to the biomass mass contribution, the interactions at high beech sawdust proportions actually inhibit tar formation relative to theoretical mixing. It is speculated that the abundant reactive radicals released from biomass at high blending ratios promote the secondary cracking of heavy tar components into non-condensable gases, which is also consistent with the synergistically increased gas yields observed (as shown in Table 2).

**Figure 5:**
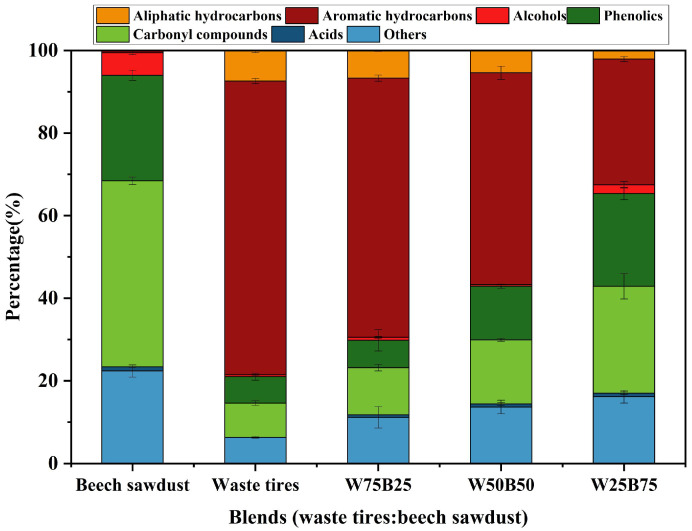
Distribution of tar components.

Figure 5 shows the compositional distribution of tars collected from waste tires, beech sawdust, and their blends in the fixed-bed reactor. Significant differences in tar composition were observed between waste tires and beech sawdust. Waste tires tar was dominated by hydrocarbons, with aromatic hydrocarbons accounting for 71.15% and aliphatic hydrocarbons for 7.42% (total 78.57%) [7]. In contrast, beech sawdust tar was dominated by oxygenated compounds (77.07%), with carbonyl compounds at 45.08% and phenolics at 25.59%, while aromatic hydrocarbons accounted for only 0.57% and aliphatic hydrocarbons were nearly undetectable [37,38].

In co-pyrolysis, increasing beech sawdust content (from waste tires to W75B25, W50B50, and W25B75) led to systematic changes in tar composition. The relative content of total hydrocarbons decreased continuously, with aromatics declining from 71.15% (waste tires) to 62.79% (W75B25), 51.31% (W50B50), and 30.41% (W25B75). Aliphatic hydrocarbons followed a similar trend, decreasing from 7.42% (waste tires) to 6.69%, 5.41%, and 2.12% for W75B25, W50B50, and W25B75, respectively. Meanwhile, oxygenated compounds from biomass increased significantly: phenolics rose from 6.39% to 22.48%, and carbonyls from 8.28% to 25.88%. Acid content remained low but slightly increased (0% to 0.87%), while alcohol content showed a non-monotonic increase, overall rising from 0.46% to 2.12%, with minor fluctuations at intermediate blending ratios. The “Others” category also increased steadily from 6.30% to 16.12%, with higher beech sawdust content. These trends indicate that co-pyrolysis alters the formation of specific components, potentially via synergistic effects.

**Figure 6:**
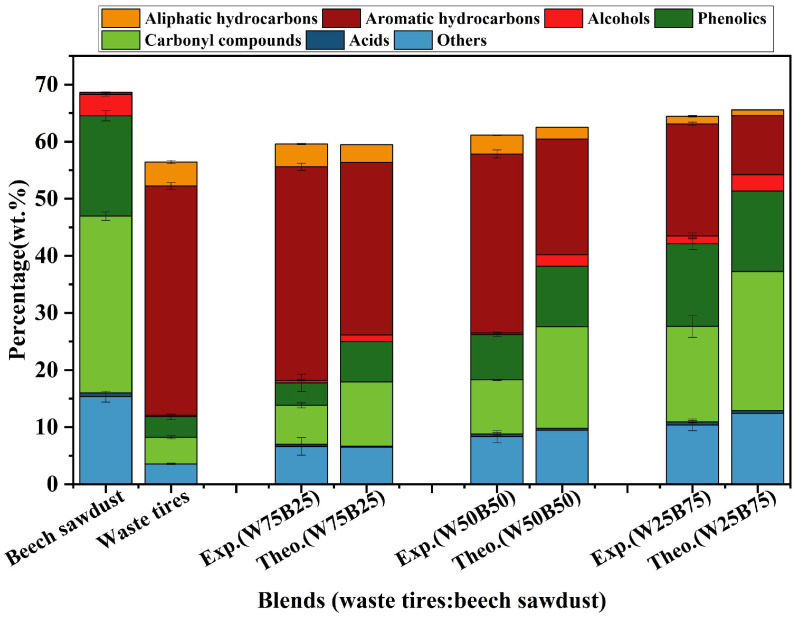
Absolute contents of different components in tar.

Figure 6 presents the absolute yields of each component. Beech sawdust-derived tar mainly consisted of oxygenated compounds, with carbonyls at 30.94 wt.%, phenolics at 17.56 wt.%, and hydrocarbons nearly negligible. In contrast, waste tires pyrolysis produced predominantly hydrocarbons: aromatic hydrocarbons being 40.14 wt.% and aliphatic hydrocarbons being 4.19 wt.%, totaling 44.33 wt.%, while oxygenated compounds were only 8.54 wt.%. In co-pyrolysis, increasing beech sawdust content systematically decreased hydrocarbon yields (e.g., aromatics from 40.14 wt.% in waste tires to 19.60 wt.% in W25B75) and increased oxygenated compounds, including phenolics (3.61 wt.% to 14.49 wt.%), carbonyls (4.67 wt.% to 16.68 wt.%), and alcohols (0.26 wt.% to 1.37 wt.%, with fluctuations at W50B50).

Comparison with theoretical values revealed that total aromatic and aliphatic hydrocarbon yield consistently exceeded theoretical predictions [17,35]. For W50B50, aromatic hydrocarbons reached 31.37 wt.% vs. 20.27 wt.% theoretically, a synergistic increase of 54.76%; aliphatic hydrocarbons reached 3.30 wt.% vs. 2.09 wt.% theoretically. It demonstrates a positive interaction between waste tires and beech sawdust during co-pyrolysis, likely involving hydrogen transfer mechanisms that promote hydrocarbon formation. Conversely, certain oxygenated compounds, particularly carbonyls and phenolics, were generally lower than theoretical values, indicating reduced formation of less stable components and improved tar quality (e.g., carbonyls in W25B75: 16.68 wt.% vs. 24.37 wt.% theoretical; phenolics in W75B25: 3.95 wt.% vs. 7.10 wt.% theoretical). Hence, the co-pyrolysis not only enhanced hydrocarbon yields via synergistic effects, which potentially increasing tar calorific value, but also suppressed oxygenated compounds, thus also effectively improving overall tar quality [19,39].

**Figure 7:**
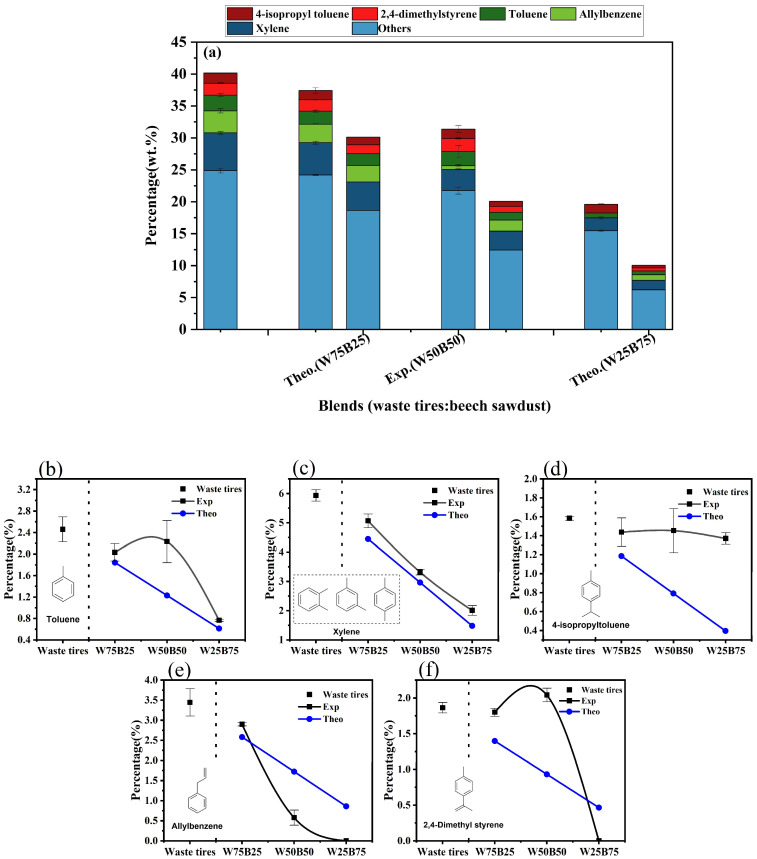
Contents of individual aromatic hydrocarbon components: (**a**) major aromatic hydrocarbons; (**b**) toluene; (**c**) xylene; (**d**) 4-isopropyl toluene; (**e**) allylbenzene; (**f**) 2,4-dimethylstyrene.

Figure 7 illustrates the main aromatic hydrocarbon components. Waste tires pyrolysis generated a large number of aromatics (40.14 wt.%), while beech sawdust produced very few (0.39 wt.%). The total experimental aromatic yield during co-pyrolysis decreased with increasing beech sawdust content: 37.42 wt.% (W75B25), 31.37 wt.% (W50B50), and 19.60 wt.% (W25B75), remaining significantly above theoretical values (30.20%, 20.26%, and 10.33%), indicating a positive synergistic effect in aromatic formation. Major aromatics detected in waste tires tar included xylenes (5.93 wt.%), allylbenzene (3.44 wt.%), toluene (2.46 wt.%), 2,4-dimethylstyrene (1.86 wt.%), and 4-isopropyl toluene (1.58 wt.%). Co-pyrolysis led to distinct trends: xylenes decreased monotonically from 5.93 wt.% to 2.01 wt.% (W25B75) and allylbenzene decreased from 3.44 wt.% (waste tires) to 2.90 wt.% (W75B25), 0.58 wt.% (W50B50), and was undetectable in W25B75. Toluene showed non-monotonic changes, first decreasing to 2.03 wt.% (W75B25), increasing to 2.23 wt.% (W50B50), then decreasing to 0.77 wt.% (W25B75). 2,4-Dimethylstyrene exhibited similar trends, with a peak at W50B50 (2.04 wt.%) before disappearing in W25B75. In contrast, 4-isopropyl toluene remained relatively stable (waste tires: 1.58 wt.%; W75B25: 1.44 wt.%; W50B50: 1.45 wt.%; W25B75: 1.37 wt.%).

Comparison with theoretical values indicates that xylenes, toluene, and 4-isopropyl toluene consistently exceeded predictions, suggesting that co-pyrolysis promotes the formation or preservation of thermally stable alkylbenzenes. Unsaturated aromatics such as allylbenzene and 2,4-dimethylstyrene exhibited complex synergistic behavior: at low beech sawdust ratios, their formation was promoted (e.g., allylbenzene in W75B25 was 12.4% above theoretical), whereas at high beech sawdust ratios, their yields were significantly suppressed or completely eliminated, reducing unstable components and enhancing tar stability.

#### 3.3.2. Gas Analysis

Figure 3 shows that the total gas yield from the pyrolysis of waste tires alone was only 2.92 wt.%, whereas beech sawdust alone produced a much higher gas yield of 12.06 wt.%. During co-pyrolysis, the gas yield increased continuously with higher beech sawdust blending ratios, rising from 2.92 wt.% for waste tires to 10.72 wt.% for W25B75. Notably, the experimental gas yields at all blending ratios exceeded the theoretical values, indicating a promoting effect of co-pyrolysis on total gas generation. For instance, at the W50B50 blending ratio, the experimental gas yield reached 8.36 wt.%, surpassing the theoretical value of 7.49 wt.%.

**Figure 8:**
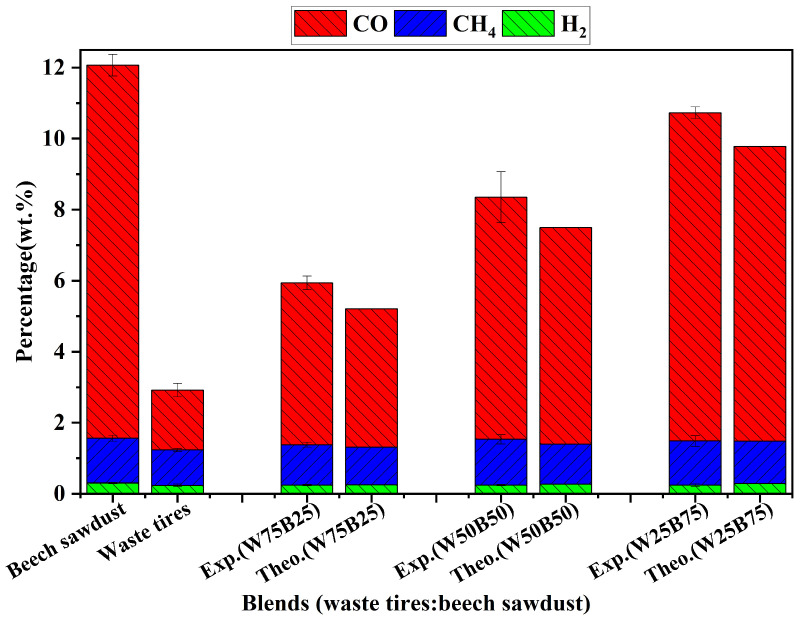
Yields of major pyrolysis gas components from waste tires, beech sawdust, and their blends (W75B25, W50B50, W25B75).

Regarding the main gas components, as shown in Figure 8, H_2_, CH_4_, and CO were the primary pyrolysis gases. It should be noted that although CO_2_ is inherently a major gaseous product of biomass pyrolysis, it could not be reliably quantified in this study because the specific micro-GC column used for the separation of light gases completely adsorbed the CO_2_. For waste tires alone, the yields of H_2_, CH_4_, and CO were 0.23 wt.%, 1.00 wt.%, and 1.69 wt.%, respectively, while beech sawdust produced 0.30 wt.%, 1.26 wt.%, and 10.50 wt.%, respectively. In co-pyrolysis, the experimental yield of CO increased steadily with beech sawdust content, from 1.69 wt.% (waste tires) to 9.24 wt.% (W25B75). CH_4_ exhibited a non-monotonic trend, initially increasing and reaching a maximum of 1.29 wt.% at W50B50, and remaining relatively high at 1.24 wt.% in W25B75. In contrast, H_2_ yields remained relatively stable, fluctuating around 0.24 wt.%.

Comparison of experimental and theoretical values shows that CH_4_ and CO yields exceeded theoretical predictions for all blending ratios. At W50B50, CH_4_ was 13.9% higher than the theoretical value, while CO was 11.9% higher, indicating that co-pyrolysis promotes the formation of these two gases. The increase in CO is consistent with recent co-pyrolysis literature [29], which may be driven by the enhanced decarbonylation of intermediate oxygenates (such as carbonyls and furans). While the cited literature reported a negative synergistic effect for CH_4_, a positive synergy was observed in this study. This difference might be attributed to the specific experimental conditions, under which the cross-interactions between the feedstocks could have promoted the demethylation of methoxy groups in beech sawdust lignin and the secondary cracking of aliphatic side chains from waste tires, thereby contributing to CH_4_ formation. Conversely, in agreement with the aforementioned literature [29], the experimental H_2_ yield was slightly lower than the theoretical value across all blending ratios, and this slight decrease falls outside the range of experimental error; at W50B50, it was 9.4% below the theoretical value, suggesting a minor inhibitory effect on H_2_ formation. It may be attributed to the promotion of certain hydrogen-consuming reactions or partial suppression of hydrogen-generating pathways during co-pyrolysis.

#### 3.3.3. Char Analysis

**Figure 9:**
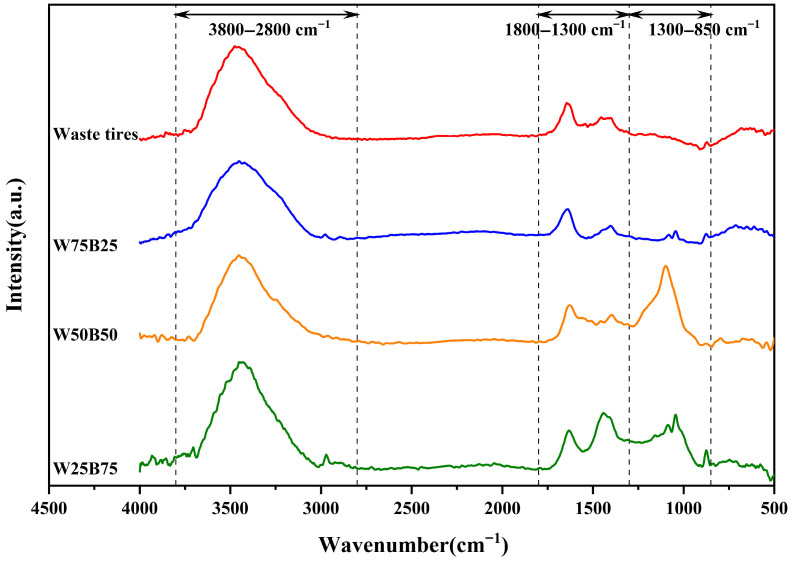
FTIR spectra of pyrolysis char with different beech sawdust blending ratios.

Figure 9 shows the FTIR spectra of pyrolysis char obtained from different beech sawdust blending ratios. Based on the evolution of functional groups, the spectra can be mainly divided into three regions: 3800–2800 cm^−1^, 1800–1300 cm^−1^, and 1300–850 cm^−1^.

**Figure 10:**
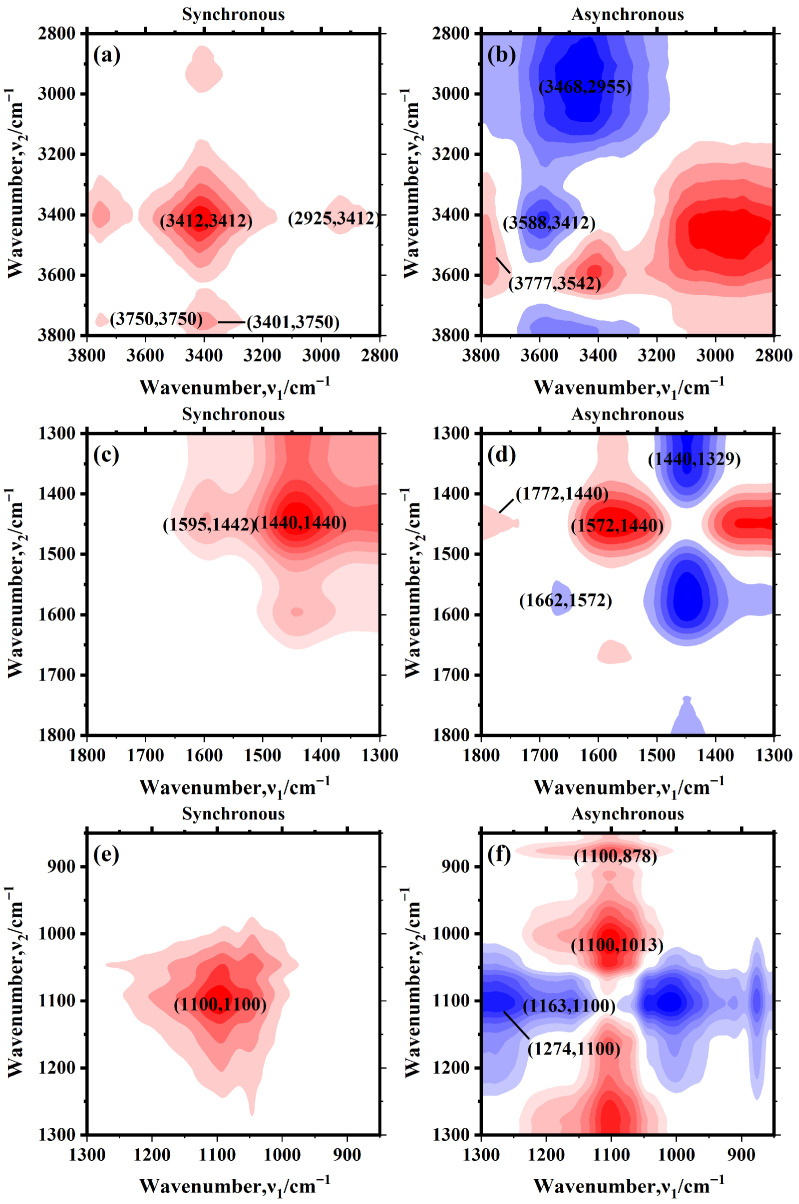
2D-COS spectra of FTIR for chars with different beech sawdust blending ratios: (**a**,**c**,**e**) synchronous spectra and (**b**,**d**,**f**) asynchronous spectra (red/blue: positive/negative correlation value, color intensity frow light to dark: correlation strength from low to high).

Two-dimensional correlation spectroscopy (2D-COS) was applied to further analyze the IR data, and the results are presented in Figure 10, with corresponding functional groups summarized in Table 3.

In the 3800–2800 cm^−1^ region, signals are mainly attributed to stretching vibrations of hydroxyl (O-H) and aliphatic (C-H) groups. As shown in Figure 10a, a strong positive auto-peak appears at 3412 cm^−1^, and a weak positive auto-peak at 3750 cm^−1^, along with two positive cross-peaks at (3401, 3750) cm^−1^ and (2925, 3412) cm^−1^. It indicates that the alcohol OH groups are the most sensitive to changes in the beech sawdust blending ratio. Additionally, the free OH groups and alkane CH groups exhibited trends similar to those of the alcohol OH groups. In Figure 10b, two negative cross-peaks at (3468, 2955) cm^−1^ and (3588, 3412) cm^−1^ and one positive cross-peak at (3777, 3542) cm^−1^ were observed. The trend of variation with increasing beech sawdust content follows as: alkane CH group > free OH group > alcohol OH group. It may be attributed to the promotion of long-chain cleavage of waste tires by beech sawdust addition, affecting the alkane C-H content in char.

In the 1800–1300 cm^−1^ region, signals are dominated by stretching vibrations of unsaturated bonds and bending vibrations of C-H. In Figure 10c, a positive auto-peak appears at 1440 cm^−1^ along with a positive cross-peak at (1595, 1442) cm^−1^, suggesting that the alkane C-H group is most sensitive to beech sawdust blending ratio changes. Additionally, the cyclic alkene C=C group exhibits a trend similar to that of the alkane CH group. In Figure 10d, two positive cross-peaks at (1772, 1440) cm^−1^ and (1572, 1440) cm^−1^ and two negative cross-peaks at (1662, 1572) cm^−1^ and (1440, 1329) cm^−1^ were observed. The overall trend of variation is as follows: 1572 cm^−1^ (cyclic alkene C=C) > 1772 cm^−1^ (conjugated acid C=O) > 1329 cm^−1^ (phenol OH) > 1662 cm^−1^ (alkene C=C) > 1440 cm^−1^ (alkane CH). It suggests that beech sawdust addition promotes depolymerization and aromatization of waste tires during pyrolysis.

In the 1300–850 cm^−1^ region, a positive auto-peak at 1100 cm^−1^ in Figure 10e indicates that the secondary alcohol OH group is most sensitive to beech sawdust content changes. In Figure 10f, two positive cross-peaks at (1100, 878) cm^−1^ and (1100, 1013) cm^−1^, along with two negative cross-peaks at (1274, 1100) cm^−1^ and (1163, 1100) cm^−1^, were observed. The trend of variation with increasing beech sawdust content is: 1100 cm^−1^ (secondary alcohol C-O) > 1163 cm^−1^ (ester C-O) > 1013 cm^−1^ (primary alcohol C-O) > 1274 cm^−1^ (aromatic ester C-O) > 878 cm^−1^ (1,2,4-trisubstituted C-H).

#### 3.3.4. Co-Pyrolysis Mechanism Analysis

Based on the above analyses, it is evident that the addition of beech sawdust exerts a significant synergistic effect on the pyrolysis of waste tires, ultimately promoting the conversion of char to volatiles, resulting in increased tar and gas yields and a decreased char yield [16,40]. The synergistic effects can be attributed to the decomposition of both feed stocks. Primarily, the scission of waste tire polymers (NR, SBR, BR) generated a pool of highly unsaturated hydrocarbon intermediates (R1) [11]. Simultaneously, the decomposition of the three main biomass components (cellulose, hemicellulose, lignin) produces not only various oxygenated compounds (R2) [41] but also amounts of highly reactive hydrogen and methyl radicals [42,43].

Notably, the synergistic effect varies across different temperature ranges. From the initial stage of pyrolysis to 384 °C, beech sawdust addition inhibits waste tires pyrolysis. For instance, at 384 °C, the residual weight of W50B50 is 57.03%, significantly higher than the theoretical value of 50.64%. It may be attributed to physical heat transfer limitations: from a heat transfer perspective, the main reason is that softened waste tires adhere to portions of beech sawdust, hindering its decomposition. As pyrolysis progresses, in the temperature range of 384–440 °C, beech sawdust addition promotes waste tires pyrolysis, as evidenced by the noticeable decrease in char yield with increasing beech sawdust content, while tar and gas yields correspondingly increase. Fixed-bed reactor experiments at 500 °C further confirm this trend: the char yield decreased from 40.67 wt.% for pure waste tires to 24.83 wt.% for the W25B75 blend, while tar and gas yields increased from 56.41 wt.% and 2.92 wt.% to 64.45 wt.% and 10.72 wt.%, respectively.

Regarding the tar composition, a key finding is the increase in aromatic hydrocarbons and the inhibition of oxygenated compounds relative to theoretical values. It is hypothesized that the radicals from beech sawdust can act catalytically in the transformation of highly unsaturated hydrocarbons, potentially promoting cyclization and aromatization (R3) [44,45,46]. Additionally, the conversion of oxygenated compounds offers another plausible route to aromatics. As a working interpretation consistent with the observed product distribution, phenolics derived from beech sawdust lignin likely undergo hydrodeoxygenation in the hydrogen-rich environment during co-pyrolysis, further converting into monoaromatic hydrocarbons such as benzene and toluene. Meanwhile, other oxygenates such as furans and carbonyls originating from cellulose/hemicellulose are transformed into aromatics through oligomerization and aromatization (R4) [47].

**Figure 11:**
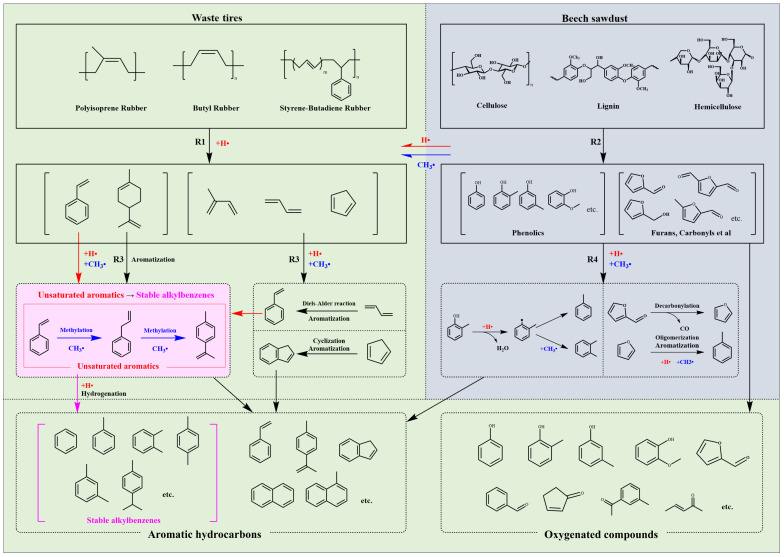
Schematic diagram of the interaction mechanism during co-pyrolysis of waste tires and beech sawdust.

Furthermore, the beech sawdust blending ratio significantly affects the aromatic hydrocarbon composition. At low beech sawdust ratios, the hydrogen sources provided by beech sawdust facilitate the formation of unsaturated aromatics, such as allylbenzene and 2,4-dimethylstyrene. As the beech sawdust ratio increases, these unsaturated aromatics undergo further hydrogenation under the influence of additional hydrogen radicals, converting into more stable alkylbenzenes. Overall, the proposed interaction mechanism during co-pyrolysis of waste tires and beech sawdust is illustrated in Figure 11.

## 4. Conclusions

This study investigates the pyrolysis behavior and synergistic mechanisms of waste tires and beech sawdust under different blending ratios. Thermogravimetric analysis reveals that the addition of beech sawdust lowered the decomposition temperature of the blends and exhibited a two-stage synergistic behavior at 384 °C. Fixed-bed pyrolysis experiments demonstrate that beech sawdust addition effectively promoted the conversion of char into volatiles, resulting in increased tar and gas yields.

The analysis of tar composition further indicates that co-pyrolysis synergistically suppressed the formation of oxygenated compounds while enhancing the production of aromatic hydrocarbons, with the aromatic hydrocarbon yield of the W50B50 blend reaching 31.37 wt.%, markedly higher than its theoretical expectation of 20.27 wt.%. Among the aromatic components, stable alkylbenzenes such as xylenes and toluene were continuously promoted, while unsaturated aromatics with side chains, including allylbenzene and 2,4-dimethylstyrene, exhibited non-monotonic synergistic behavior: promoted at low beech sawdust content but strongly inhibited at high beech sawdust content. Gas and char analyses corroborated these complex interactions, showing synergistic increases in CO and CH_4_, slight inhibition of H_2_, and evolution of functional groups in the char.

Based on these findings, a working interpretation of the synergistic mechanism for waste tires–beech sawdust co-pyrolysis is proposed: hydrogen radicals released from beech sawdust pyrolysis are hypothesized to play a key role in the cyclization and aromatization of waste tires depolymerization products, promoting the formation of stable aromatic hydrocarbons. Meanwhile, it is highly likely that some oxygenated compounds undergo hydrodeoxygenation in the hydrogen-rich environment to form monoaromatic hydrocarbons. The blending ratio influences the product distribution, as low ratios favor unsaturated aromatics, while high ratios promote their hydrogenation to stable alkylbenzenes. In summary, co-pyrolysis can optimize the composition and quality of waste tires pyrolysis products through synergistic effects.

## Figures and Tables

**Table 1:** Ultimate and proximate analyses of waste tires and beech sawdust (Air-dry basis).

Samples	Ultimate Analysis (wt.%)					Proximate Analysis (wt.%)			
	C	H	N	S	O	Moisture	Volatiles	Fixed Carbon	Ash
Waste tires	77.75	6.65	0.52	1.53	13.55	0.74	62.08	25.85	11.33
Beech sawdust	46.30	6.08	0.09	0	47.53	5.42	82.44	11.65	0.49

**Table 2:** Synergistic effect of beech sawdust addition on the product yields of blends.

Blends	ΔY_Tar_ (%)	ΔY_Char_ (%)	ΔY_Gas_ (%)
W75B25	0.20 ± 0.09	−2.39 ± 0.75	13.91 ± 2.02
W50B50	−2.22 ± 1.21	1.71 ± 1.25	11.63 ± 1.11
W25B75	−1.70 ± 0.47	0.71 ± 0.29	9.63 ± 1.46

Note: A positive value indicates a promoting synergistic effect (experimental yield > theoretical yield), while a negative value indicates an inhibitory effect.

**Table 3:** Infrared absorption peak ranges of relevant chemical bonds.

Functional Group	Peak/cm^−1^	Vibration
Free O-H	3800–3650	Stretching
Alcohol O-H	3600–3200	Stretching
Alkane C-H	3100–2840	Stretching
Conjugated acid C=O	1772	Stretching
Alkene C=C	1662	Stretching
Cyclic alkene C=C	1650–1566	Stretching
Alkane C-H	1440	Bending
Phenol O-H	1329	Bending
Aromatic ester C-O	1274	Stretching
Ester C-O	1163	Stretching

## Data Availability

The original contributions presented in this study are included in the article and Supplementary Materials. Further inquiries can be directed to the corresponding authors.

## Supplementary Materials

The following supporting information can be downloaded at: https://www.mdpi.com/article/10.3390/ma19081495/s1, Table S1: Chromatographic relative peak area (%) and qual of detected organics in tar produced from the pyrolysis of waste tires, beech sawdust and their blends; Table S2: Chromatographic concentration of detected organics (mol/10 g samples) in gas produced from the pyrolysis of waste tires and beech sawdust; Table S3: Chromatographic concentration of detected organics (mol/10 g samples) in gas produced from the pyrolysis of the blends.

## Abbreviations

The following abbreviations are used in this manuscript:

NR	Natural rubber
BR	Butadiene rubber
SBR	Styrene-butadiene rubber
GC-MS	Gas chromatography-mass spectrometry
FTIR	Fourier transform infrared spectroscopy
2D-COS	Two-dimensional correlation spectroscopy
TG&DTG	Thermogravimetry & Derivative Thermogravimetry

## References

[1] Buddhacosa N., Khatibi A., Das R., Giustozzi F., Galos J., Kandare E. (2023). Crush behaviour and vibration damping properties of syntactic foam incorporating waste tyre-derived crumb rubber. J. Mater. Res. Technol..

[2] Jiang H., Shao J., Zhu Y., Yu J., Cheng W., Yang H., Zhang X., Chen H. (2023). Production mechanism of high-quality carbon black from high-temperature pyrolysis of waste tire. J. Hazard. Mater..

[3] Siddika A., Mamun M.A.A., Alyousef R., Amran Y.H.M., Aslani F., Alabduljabbar H. (2019). Properties and utilizations of waste tire rubber in concrete: A review. Constr. Build. Mater..

[4] Wang M., Zhang L., Li A., Irfan M., Du Y., Di W. (2019). Comparative pyrolysis behaviors of tire tread and side wall from waste tire and characterization of the resulting chars. J. Environ. Manag..

[5] Czajczyńska D., Anguilano L., Ghazal H., Krzyżyńska R., Reynolds A.J., Spencer N., Jouhara H. (2017). Potential of pyrolysis processes in the waste management sector. Therm. Sci. Eng. Prog..

[6] Zheng D., Cheng J., Dai C., Xu R., Wang X., Liu N., Wang N., Yu G., Chen B. (2022). Study of passenger-car-waste-tire pyrolysis: Behavior and mechanism under kinetical regime. Waste Manag..

[7] Williams P.T. (2013). Pyrolysis of waste tyres: A review. Waste Manag..

[8] Dieguez-Alonso A., Vu-Han T.-L.E., Almuina-Villar H., Fuentes J.J.R., Hilfert L., Dernbecher A., de la Rosa J.M., Behrendt F. (2023). Tailored production and application of biochar for tar removal. Fuel.

[9] Xia C., Cao C., Cheng J., Zhang Q., Ding Y., Liu H. (2025). Thermal degradation behavior and conversion pathways of condensed nitrophenol contaminant under confined space: A kinetic and mechanistic investigation. J. Environ. Chem. Eng..

[10] Islam M.N., Nahian M.R. (2016). Improvement of Waste Tire Pyrolysis Oil and Performance Test with Diesel in CI Engine. J. Renew. Energy.

[11] Li D., Lei S., Lin F., Zhong L., Ma W., Chen G. (2020). Study of scrap tires pyrolysis—Products distribution and mechanism. Energy.

[12] Wang S., Cheng M., Xie M., Yang Y., Liu T., Zhou T., Cen Q., Liu Z., Li B. (2025). From waste to energy: Comprehensive understanding of the thermal-chemical utilization techniques for waste tire recycling. Renew. Sustain. Energy Rev..

[13] Han W., Jiang C., Wang J., Chen H. (2022). Enhancement of heat transfer during rubber pyrolysis process. J. Clean. Prod..

[14] Farooq M.Z., Zeeshan M., Iqbal S., Ahmed N., Shah S.A.Y. (2018). Influence of waste tire addition on wheat straw pyrolysis yield and oil quality. Energy.

[15] Alvarez J., Amutio M., Lopez G., Santamaria L., Bilbao J., Olazar M. (2019). Improving bio-oil properties through the fast co-pyrolysis of lignocellulosic biomass and waste tyres. Waste Manag..

[16] Shah S.A.Y., Zeeshan M., Farooq M.Z., Ahmed N., Iqbal N. (2019). Co-pyrolysis of cotton stalk and waste tire with a focus on liquid yield quantity and quality. Renew. Energy.

[17] Wang Z., Wu M., Chen G., Zhang M., Sun T., Burra K.G., Guo S., Chen Y., Yang S., Li Z. (2023). Co-pyrolysis characteristics of waste tire and maize stalk using TGA, FTIR and Py-GC/MS analysis. Fuel.

[18] Martínez J.D., Veses A., Mastral A.M., Murillo R., Navarro M.V., Puy N., Artigues A., Bartrolí J., García T. (2014). Co-pyrolysis of biomass with waste tyres: Upgrading of liquid bio-fuel. Fuel Process. Technol..

[19] Khan S.R., Zeeshan M., Khokhar M.F., Zeshan, Ahmad I. (2023). A comprehensive study on upgradation of pyrolysis products through co-feeding of waste tire into rice straw under broad range of co-feed ratios in a bench-scale fixed bed reactor. Biomass Convers. Biorefinery.

[20] Xu Q., Chen Z., Xian S., Li H., Wu Y. (2025). In-situ sulfur fixation mechanism during microwave fluidized-bed co-pyrolysis of waste tires and biomass. J. Clean. Prod..

[21] (2008). Proximate Analysis of Coal.

[22] (2012). Proximate Analysis of Solid Biofuels.

[23] Skreiberg A., Skreiberg Ø., Sandquist J., Sørum L. (2011). TGA and macro-TGA characterisation of biomass fuels and fuel mixtures. Fuel.

[24] Policella M., Wang Z., Burra K.G., Gupta A.K. (2019). Characteristics of syngas from pyrolysis and CO_2_-assisted gasification of waste tires. Appl. Energy.

[25] Wang H., Hu H., Yang Y., Liu H., Tang H., Xu S., Li A., Yao H. (2020). Effect of high heating rates on products distribution and sulfur transformation during the pyrolysis of waste tires. Waste Manag..

[26] Zhang P., Chen Z., Zhang Q., Zhang S., Ning X., Zhou J. (2022). Co-pyrolysis characteristics and kinetics of low metamorphic coal and pine sawdust. RSC Adv..

[27] Song F., Li T., Wu F., Leung K.M.Y., Hur J., Zhou L., Bai Y., Zhao X., He W., Ruan M. (2023). Temperature-Dependent Molecular Evolution of Biochar-Derived Dissolved Black Carbon and Its Interaction Mechanism with Polyvinyl Chloride Microplastics. Environ. Sci. Technol..

[28] Zhang J., Zou H., Liu J., Evrendilek F., Xie W., He Y., Buyukada M. (2021). Comparative (co-)pyrolytic performances and by-products of textile dyeing sludge and cattle manure: Deeper insights from Py-GC/MS, TG-FTIR, 2D-COS and PCA analyses. J. Hazard. Mater..

[29] Niu M., Sun R., Ding K., Gu H., Cui X., Wang L., Hu J. (2022). Synergistic effect on thermal behavior and product characteristics during co-pyrolysis of biomass and waste tire: Influence of biomass species and waste blending ratios. Energy.

[30] Shan T., Chen H., Liu T., Ma Z., Tan Y., Zhang H. (2025). Synergistic effects in the Co-pyrolysis of waste tires, plastics, and corn stalks: Kinetic and thermodynamic analyses for enhanced resource utilization. Renew. Energy.

[31] Chen D., Cen K., Zhuang X., Gan Z., Zhou J., Zhang Y., Zhang H. (2022). Insight into biomass pyrolysis mechanism based on cellulose, hemicellulose, and lignin: Evolution of volatiles and kinetics, elucidation of reaction pathways, and characterization of gas, biochar and bio-oil. Combust. Flame.

[32] Yan X., Hu J., Zhang Q., Zhao S., Dang J., Wang W. (2020). Chemical-looping gasification of corn straw with Fe-based oxygen carrier: Thermogravimetric analysis. Bioresour. Technol..

[33] Chin B.L.F., Yusup S., Al Shoaibi A., Kannan P., Srinivasakannan C., Sulaiman S.A. (2014). Kinetic studies of co-pyrolysis of rubber seed shell with high density polyethylene. Energy Convers. Manag..

[34] Arabiourrutia M., Lopez G., Artetxe M., Alvarez J., Bilbao J., Olazar M. (2020). Waste tyre valorization by catalytic pyrolysis—A review. Renew. Sustain. Energy Rev..

[35] Wang L., Chai M., Liu R., Cai J. (2018). Synergetic effects during co-pyrolysis of biomass and waste tire: A study on product distribution and reaction kinetics. Bioresour. Technol..

[36] Alzahrani N., Nahil M.A., Williams P.T. (2025). Co-pyrolysis of waste plastics and tires: Influence of interaction on product oil and gas composition. J. Energy Inst..

[37] Li S., Dong L., Hu H., Huang Y., Wang Y., Gong L., Zhang M., Xu S., Yao H. (2025). Ash characteristics during co-incineration with industrial organic solid waste in a large-scale municipal solid waste incinerator. Int. J. Coal Sci. Techn..

[38] Hansen S., Mirkouei A., Diaz L.A. (2020). A comprehensive state-of-technology review for upgrading bio-oil to renewable or blended hydrocarbon fuels. Renew. Sustain. Energy Rev..

[39] Khan S.R., Zeeshan M., Masood A. (2020). Enhancement of hydrocarbons production through co-pyrolysis of acid-treated biomass and waste tire in a fixed bed reactor. Waste Manag..

[40] Uçar S., Karagöz S. (2014). Co-pyrolysis of pine nut shells with scrap tires. Fuel.

[41] Wang S., Dai G., Yang H., Luo Z. (2017). Lignocellulosic biomass pyrolysis mechanism: A state-of-the-art review. Prog. Energy Combust. Sci..

[42] Brebu M., Tamminen T., Spiridon I. (2013). Thermal degradation of various lignins by TG-MS/FTIR and Py-GC-MS. J. Anal. Appl. Pyrolysis.

[43] Liu P., Wang Y., Zhou Z., Yuan H., Zheng T., Chen Y. (2020). Effect of carbon structure on hydrogen release derived from different biomass pyrolysis. Fuel.

[44] Li J., Zheng D., Yao Z., Wang S., Xu R., Deng S., Chen B., Wang J. (2022). Formation Mechanism of Monocyclic Aromatic Hydrocarbons during Pyrolysis of Styrene Butadiene Rubber in Waste Passenger Car Tires. ACS Omega.

[45] Ye W., Xu X., Zhan M., Huang Q., Li X., Jiao W., Yin Y. (2022). Formation behavior of PAHs during pyrolysis of waste tires. J. Hazard. Mater..

[46] Zhang Y., Li X., Xie W., Lu Y., Wang X., Zhang L., Ji G., Gao Y., Li A. (2025). Pyrolysis behavior and production characteristics of limonene in tire pyrolysis: Implications for waste valorization. Fuel.

[47] Wang K., Kim K.H., Brown R.C. (2014). Catalytic pyrolysis of individual components of lignocellulosic biomass. Green Chem..

