# Supplementary material for: Co-Pyrolysis of Waste Tires and Beech Sawdust: Comprehensive Analysis of Thermal Behavior, Synergistic Effect, and Interaction Mechanisms
Source: Materials (Basel). 2026 Apr 8;19(8):1495. doi: 10.3390/ma19081495 (PMC13117236; doi:10.3390/ma19081495)
Supplement: Supplementary file 1 [file materials-19-01495-s001.zip › materials-4208883-supplementary.pdf]

Supplementary Materials

Co-Pyrolysis of Waste Tires and Beech Sawdust: Comprehensive Analysis of Thermal Behavior, Synergistic Effect, and Interaction Mechanisms

Guangyao Zheng [1], Chengyang Cao [1,*], Qiming Zhang [1,*], Pei Jia [1], Lu Dong [2] and Hongyun Hu [3]

[1] School of Resource & Safety Engineering, Wuhan Institute of Technology, Wuhan 430074, China
[2] State Key Laboratory of Low Carbon Catalysis and Carbon Dioxide Utilization, School of Petroleum Engineering, Yangtze University, Wuhan 430100, China
[3] State Key Laboratory of Coal Combustion, Huazhong University of Science and Technology, Wuhan 430074, China
* Correspondence: ccy9311@wit.edu.cn (C.C.); zhangqiming2736@163.com (Q.Z.)

Contents

Contents ....……….....1

Table S1. Chromatographic relative peak area (%) and qual of detected organics in tar produced from the pyrolysis of Waste tires, Beech sawdust and their blends. 2

Table S2. Chromatographic concentration of detected organics (mol/ 10g samples) in gas produced from the pyrolysis of Waste tires and Beech sawdust. 9

Table S3. Chromatographic concentration of detected organics (mol/ 10g samples) in gas produced from the pyrolysis of the Blends.9

Table S1. Chromatographic relative peak area (%) and qual of detected organics in tar produced from the pyrolysis of Waste tires, Beech sawdust and their blends.

Tar Species	CAS	RT (min)	Waste Tires	Beech Sawdust	W75B25	W50B50	W25B75
2,2,4-Trimethyl-1,3-dioxolane	001193-11-9	3.2117	0.0000	0.0000	0.3588(80)	0.4074(80)	0.0000
Thioacetic acid	000507-09-5	3.3008	0.0000	0.3651(53)	0.0000	0.0000	0.0000
2,3-Dimethyl-2-butene	000563-79-1	3.485	0.6188(80)	0.0000	0.0000	0.0000	0.0000
3-Penten-2-one, (E)-	003102-33-8	3.4907	0.0000	0.0000	0.9608(87)	0.8382(90)	0.0000
1-Cyclopropyl-ethanone	000765-43-5	3.5026	0.0000	0.4988(81)	0.0000	0.0000	0.978(87)
2-Butenal, 2-methyl-	001115-11-3	3.5502	0.4434(64)	0.0000	0.4973(87)	0.0000	0.3918(58)
2-Butenal, 2-methyl-, (E)-	000497-03-0	3.5561	0.0000	0.0000	0.0000	0.3825(87)	0.0000
1,4-Cyclohexadiene, 1-methyl-	004313-57-9	3.7222	0.0000	0.0000	0.3121(95)	0.0000	0.0000
2,3-Butanedione, mono(O-methyloxime)	000617-32-3	3.7757	0.0000	0.0000	0.7364(64)	0.4586(59)	0.0000
Toluene	000108-88-3	4.0486	4.3568(94)	0.0000	3.4103(94)	3.6509(95)	1.1888(83)
1-Hydroxy-2-butanone	005077-67-8	4.0545	0.0000	0.9155(52)	0.0000	0.0000	0.0000
Acetic acid, methyl ester	000079-20-9	4.1378	0.0000	3.6369(53)	0.0000	1.3204(58)	2.6911(53)
Thiophene, 3-methyl-	000616-44-4	4.1557	0.3142(90)	0.0000	0.0000	0.0000	0.0000
Ethanamine, N-ethyl-	000109-89-7	4.3335	0.0000	1.8592(64)	0.0000	0.0000	0.0000
2-Penten-1-amine, N,N,2-trimethyl-, (E)-	055630-70-1	4.3455	0.0000	0.0000	0.0000	0.6229(50)	0.0000
Cyclopentene, 3-ethenyl-	026727-45-7	4.3693	0.3188(94)	0.0000	0.0000	0.0000	0.0000
2-Butenal, 3-methyl-	000107-86-8	4.4762	0.3988(90)	0.0000	0.5248(87)	0.0000	0.8351(93)
Furan, 2,3-dihydro-4-methyl-	034314-83-5	4.488	0.0000	0.0000	0.0000	0.6871(64)	0.0000
Butanoic acid	000107-92-6	4.5472	0.0000	0.5703(72)	0.0000	0.0000	0.0000
Propanoic acid, 2-oxo-, methyl ester	000600-22-6	4.6065	0.0000	0.0000	0.7506(50)	0.0000	0.0000
1,3-Dimethyl-1-cyclohexene	002808-76-6	5.5327	0.408(91)	0.0000	0.3035(87)	0.0000	0.0000
Crotonic acid	003724-65-0	5.6571	0.0000	0.4107(81)	0.0000	0.0000	0.0000
Furfural	000098-01-1	5.7225	0.0000	6.7073(93)	0.0000	2.3289(90)	5.2522(70)
2-Cyclopenten-1-one	000930-30-3	5.7345	0.5731(90)	0.0000	1.621(64)	0.0000	0.0000
2-Cyclopenten-1-one, 3-methyl-	002758-18-1	5.9481	0.0000	0.0000	0.0000	0.5858(50)	2.6213(62)
2-Pentanone, 4-hydroxy-4-methyl-	000123-42-2	6.031	0.4758(72)	0.7347(90)	0.7454(72)	0.732(59)	2.1781(90)
Maleic anhydride	000108-31-6	6.3517	0.0000	0.0000	0.0000	0.0000	0.3332(78)
2-Furanmethanol	000098-00-0	6.4465	0.0000	3.8537(95)	0.0000	0.0000	0.0000
1,6:2,3-Dianhydro-4-O-acetyl-.beta.-d-allopyranose	1000139-89-9	6.4585	0.0000	0.0000	0.0000	0.0000	1.7614(52)
Ethylbenzene	000100-41-4	6.5772	1.353(93)	0.0000	0.9581(93)	0.4756(93)	0.0000
Hexanoic acid, 3-hydroxy-5-methyl-, methyl ester	1000153-12-6	6.6544	0.0000	0.0000	0.0000	0.0000	1.8933(59)

Tar Species	CAS	RT (min)	Waste Tires	Beech Sawdust	W75B25	W50B50	W25B75
3-Azabutyl-1-ol, 4-cyclopropyl-3,3-dimethyl-, bromide	1000124-71-0	6.8145	0.0000	0.3507(53)	0.0000	0.0000	0.0000
p-Xylene	000106-42-3	6.8146	0.0000	0.0000	6.8824(97)	4.3618(97)	2.4998(97)
2-Propanone, 1-(acetyloxy)-	000592-20-1	6.868	0.0000	1.488(72)	0.0000	0.6026(52)	1.2842(72)
Cyclohexane, 1,4-bis(methylene)-	004982-20-1	7.0165	0.3901(90)	0.0000	0.0000	0.0000	0.0000
(E,E,E)-2,4,6-Octatriene	015192-80-0	7.0222	0.0000	0.0000	0.3213(93)	0.0000	0.0000
4-Cyclopentene-1,3-dione	000930-60-9	7.0698	0.0000	1.6281(96)	0.0000	0.4234(62)	1.2383(93)
Sorbic Acid	000110-44-1	7.4082	0.3946(60)	0.0000	0.0000	0.0000	0.0000
Styrene	000100-42-5	7.4557	1.7687(97)	0.0000	2.1557(97)	1.551(97)	0.0000
Bicyclo[4.2.0]octa-1,3,5-triene	000694-87-1	7.4616	0.0000	0.0000	0.0000	0.0000	1.0691(96)
Benzene, 1,3-dimethyl-	000108-38-3	7.5269	0.0000	0.0000	0.0000	1.0668(95)	0.0000
o-Xylene	000095-47-6	7.5328	10.03(97)	0.0000	1.6155(95)	0.0000	0.6162(95)
2-Cyclopenten-1-one, 2-methyl-	001120-73-6	7.9305	0.5639(90)	0.5868(91)	0.9527(89)	1.0105(90)	1.1585(93)
Butyrolactone	000096-48-0	8.0728	0.0000	1.1046(90)	0.0000	0.0000	0.9056(91)
2(5H)-Furanone	000497-23-4	8.1145	0.0000	3.2119(81)	0.0000	0.7369(72)	1.9243(81)
2-Cyclohexen-1-ol	000822-67-3	8.2747	0.0000	1.1815(53)	0.0000	0.3913(52)	0.0000
2-Cyclopenten-1-one, 2-hydroxy-	010493-98-8	8.4705	0.0000	2.6061(83)	0.0000	0.0000	0.0000
1,2-Cyclopentanedione	003008-40-0	8.4706	0.0000	0.0000	0.4443(64)	1.1217(72)	1.7512(86)
Benzene, 1-ethyl-4-methyl-	000622-96-8	8.5419	0.4251(58)	0.0000	0.5519(94)	0.0000	0.0000
2-Cyclohexen-1-one	000930-68-7	8.7257	0.0000	0.0000	0.3298(64)	0.0000	0.0000
2(5H)-Furanone, 5-methyl-	000591-11-7	8.9751	0.0000	0.5479(94)	0.0000	0.0000	0.4864(93)
2,5-Furandione, dihydro-3-methylene-	002170-03-8	9.0938	0.0000	0.5422(90)	0.0000	0.0000	0.3221(91)
1,3,6-Heptatriene, 2,5,6-trimethyl-	042123-66-0	9.2363	0.6447(60)	0.0000	0.0000	0.0000	0.0000
Benzaldehyde	000100-52-7	9.622	1.5094(91)	0.4192(96)	1.6156(95)	1.3908(95)	1.0352(96)
Benzene, 1-ethyl-3-methyl-	000620-14-4	9.7347	2.0643(94)	0.0000	1.7354(94)	0.0000	0.0000
Benzene, 1-ethyl-2-methyl-	000611-14-3	9.7764	2.6982(94)	0.0000	2.1178(92)	1.5958(95)	0.0000
2-Furancarboxaldehyde, 5-methyl-	000620-02-0	9.7822	0.0000	0.8098(76)	0.0000	0.0000	0.0000
2H-Pyran-2-one	000504-31-4	9.9543	0.0000	1.314(95)	0.0000	0.0000	1.2364(94)
Benzene, 1,2,3-trimethyl-	000526-73-8	9.9603	0.6481(97)	0.0000	0.6984(95)	0.0000	0.0000
Benzene, 1,2,4-trimethyl-	000095-63-6	9.9604	0.0000	0.0000	0.0000	0.8554(86)	0.0000
.alpha.-Methylstyrene	000098-83-9	10.3876	1.0012(86)	0.0000	0.965(91)	0.7452(89)	0.3285(50)
Phenol	000108-95-2	10.4531	1.5068(94)	1.2485(97)	1.8358(94)	2.1899(95)	1.9335(97)
2-Hydroxy-gamma-butyrolactone	019444-84-9	10.5063	0.0000	0.5059(72)	0.0000	0.0000	0.0000
Benzenemethanol, 4-hydroxy-	000623-05-2	10.6549	0.3091(53)	0.0000	0.0000	0.0000	0.0000

Tar Species	CAS	RT (min)	Waste Tires	Beech Sawdust	W75B25	W50B50	W25B75
Benzene, 2-propenyl-	000300-57-2	10.7558	6.1047(83)	0.0000	4.8686(91)	0.9436(56)	0.0000
Benzene, 1-propenyl-	000637-50-3	10.7676	0.0000	0.0000	0.0000	4.0976(92)	2.3518(95)
2-Methyliminoperhydro-1,3-oxazine	126442-19-1	10.8268	0.0000	0.7835(64)	0.0000	0.0000	0.0000
Benzene, 1-ethenyl-3-methyl-	000100-80-1	10.8743	1.8542(96)	0.0000	1.8409(96)	1.5766(96)	0.0000
Benzene, 1-ethenyl-2-methyl-	000611-15-4	10.8744	0.0000	0.0000	0.0000	0.0000	1.044(94)
Allylidenecyclohexane	005664-10-8	11.3315	0.7537(58)	0.0000	0.4386(52)	0.0000	0.0000
2-Cyclohexen-1-one, 3,4-dimethyl-	1000197-00-4	11.3967	0.0000	0.0000	0.0000	0.0000	0.3483(62)
1-Nonen-4-yne	031508-12-0	11.5689	0.0000	0.0000	0.0000	0.6633(60)	0.0000
Mesitylene	000108-67-8	11.646	2.2894(97)	0.0000	1.9504(97)	1.4309(97)	0.7526(97)
2-Cyclopenten-1-one, 2-hydroxy-3-methyl-	000080-71-7	11.7409	0.0000	1.6493(97)	0.0000	0.0000	0.0000
p-Cymene	000099-87-6	11.7529	2.8063(95)	0.0000	2.7754(93)	2.3764(93)	2.1262(90)
D-Limonene	005989-27-5	11.9012	4.0256(99)	0.0000	4.5677(99)	3.5994(99)	1.8591(99)
2H-Pyran-2-one, 5,6-dihydro-	003393-45-1	11.9723	0.0000	0.3853(58)	0.0000	0.0000	0.0000
Benzene, cyclopropyl-	000873-49-4	12.0436	0.0000	0.0000	0.6984(50)	0.0000	0.0000
1,5-Cyclooctadiene, 1-ethyl-	016539-10-9	12.1445	0.492(58)	0.0000	0.5572(70)	0.0000	0.0000
2-Cyclopenten-1-one, 2,3-dimethyl-	001121-05-7	12.1446	0.0000	0.3396(64)	0.0000	0.5631(76)	0.536(76)
Benzaldehyde, 2-hydroxy-	000090-02-8	12.2574	0.0000	1.8473(95)	0.0000	0.0000	1.2072(96)
Indene	000095-13-6	12.2989	1.2823(95)	0.0000	1.4884(94)	1.7232(94)	0.0000
Oxirane, 2,2-dimethyl-3-propyl-	017612-35-0	12.5778	0.0000	0.3525(53)	0.0000	0.0000	0.0000
Benzene, 1-methyl-3-propyl-	001074-43-7	12.6017	0.5198(94)	0.0000	0.4527(93)	0.4167(94)	0.0000
Phenol, 2-methyl-	000095-48-7	12.7322	1.1744(96)	1.0346(97)	1.3929(96)	1.572(96)	1.9419(97)
o-Cymene	000527-84-4	12.8272	0.4888(95)	0.0000	0.0000	0.0000	0.0000
Benzaldehyde, 2-methyl-	000529-20-4	12.9874	1.117(97)	0.0000	0.3831(90)	1.4779(64)	0.0000
1-Octanone, 1-phenyl-	001674-37-9	12.9875	0.329(59)	0.0000	0.0000	0.0000	0.0000
Benzaldehyde, 3-methyl-	000620-23-5	13.0468	0.0000	0.0000	1.2103(96)	0.0000	0.9255(96)
Bicyclo[3.3.0]oct-2-en-6-one, 3-methyl-	1000155-37-0	13.2366	0.0000	0.0000	0.414(91)	0.0000	0.0000
6,8-Nonadien-2-one	077828-54-7	13.2367	0.0000	0.0000	0.0000	0.59(80)	0.0000
2,5-Furandicarboxaldehyde	000823-82-5	13.3019	0.0000	0.7819(87)	0.0000	0.0000	0.8649(64)
Phenol, 3-methyl-	000108-39-4	13.391	1.7118(96)	0.8211(96)	1.953(96)	1.9756(96)	2.0338(96)
Benzene, 1-methyl-3-(1-methylethyl)-	000535-77-3	13.4503	1.4201(76)	0.0000	1.3844(81)	1.1594(64)	0.0000
Bicyclo[4.2.0]octa-1,3,5-trien-7-ol	035447-99-5	13.4504	0.0000	0.0000	0.0000	0.0000	1.0032(92)
Benzene, 1-methyl-3-(1-methylethenyl)-	001124-20-5	13.5691	0.4059(97)	0.0000	0.3748(97)	0.3655(97)	0.0000
Benzene, 1-methyl-4-(2-propenyl)-	003333-13-9	13.6461	0.0000	0.0000	0.4868(64)	0.0000	0.0000

Tar Species	CAS	RT (min)	Waste Tires	Beech Sawdust	W75B25	W50B50	W25B75
1-Phenyl-1-butene	000824-90-8	13.6462	0.0000	0.0000	0.0000	0.3904(83)	0.0000
1-Methyl-2-phenylcyclopropane	003145-76-4	13.6463	0.5369(83)	0.0000	0.0000	0.0000	0.0000
Phenol, 2-methoxy-	000090-05-1	13.7412	0.0000	4.3342(83)	0.0000	0.0000	1.6893(91)
1-methyl-4-(1-methylethenyl)-Benzene	001195-32-0	13.7768	3.3026(96)	0.0000	3.3132(96)	3.3385(96)	0.0000
1-Undecene	000821-95-4	13.9312	0.3673(95)	0.0000	0.0000	0.0000	0.0000
1,3-Benzenediol, 4,5-dimethyl-	000527-55-9	13.9963	0.0000	0.0000	0.4008(50)	0.0000	0.0000
Benzofuran, 7-methyl-	017059-52-8	13.9965	0.3792(60)	0.0000	0.0000	0.0000	0.0000
2,6,6-Trimethylbicyclo[3.2.0]hept-2-en-7-one	004613-37-0	14.0796	0.2831(87)	0.0000	0.3428(87)	0.0000	0.0000
Benzene, 2-ethenyl-1,3-dimethyl-	002039-90-9	14.1328	0.8289(60)	0.0000	0.826(92)	0.0000	0.0000
p-(1-Propenyl)-toluene	1000429-54-9	14.1389	0.0000	0.0000	0.0000	0.6496(83)	0.0000
1H-Indazole, 3-methyl-	1000316-00-2	14.1448	0.0000	0.0000	0.0000	0.0000	0.5584(60)
Phenol, 2,5-dimethyl-	000095-87-4	14.2634	2.7494(93)	0.4486(94)	0.0000	0.0000	0.0000
Phenol, 2,6-dimethyl-	000576-26-1	14.2636	0.8383(93)	0.0000	0.8758(96)	0.7807(96)	1.0628(98)
Maltol	000118-71-8	14.394	0.0000	0.3838(96)	0.0000	0.0000	0.0000
Benzene, 1-methyl-4-(1-methylpropyl)-	001595-16-0	14.406	0.3588(87)	0.0000	0.0000	0.0000	0.0000
Benzene, 2-ethenyl-1,3,5-trimethyl-	000769-25-5	14.501	0.4006(60)	0.0000	0.0000	0.3682(90)	0.0000
2H-Pyran-3(4H)-one, dihydro-6-methyl-	043152-89-2	14.6136	0.0000	1.0022(50)	0.0000	0.0000	0.0000
Benzene, (3-methyl-2-butenyl)-	004489-84-3	14.6196	0.0000	0.0000	0.0000	0.381(64)	0.0000
Benzene, 1,2,4,5-tetramethyl-	000095-93-2	14.7027	0.6147(95)	0.0000	0.5461(96)	0.4611(95)	0.3673(94)
Benzene, 2-ethenyl-1,4-dimethyl-	002039-89-6	14.9996	0.9135(96)	0.0000	0.5461(96)	0.5539(96)	0.0000
Benzaldehyde, 2-hydroxy-6-methyl-	018362-36-2	15.035	0.0000	0.4088(96)	0.0000	0.0000	0.0000
Benzaldehyde, 2-hydroxy-4-methyl-	000698-27-1	15.0351	0.0000	0.0000	0.0000	0.0000	0.7341(90)
Phenol, 2-ethyl-	000090-00-6	15.2783	0.0000	0.3683(93)	0.0000	0.0000	0.0000
Phenol, 3,5-dimethyl-	000108-68-9	15.2785	0.0000	0.0000	0.0000	0.0000	0.9358(91)
1-Penten-3-yne, 2-methyl-	000926-55-6	15.3436	0.0000	0.0000	0.7271(80)	0.0000	0.0000
Bicyclo[2.2.2]oct-2-ene	000931-64-6	15.3438	0.0000	0.0000	0.0000	0.7386(72)	0.0000
cis-8-Methyl-bicyclo(4,3,0)non-3,7-diene	1000144-50-6	15.3438	0.6795(80)	0.0000	0.0000	0.0000	0.0000
trans-8-Methyl-bicyclo[4.3.0]non-3,7-diene	1000144-50-7	15.4447	0.3136(58)	0.0000	0.0000	0.0000	0.0000
Phenol, 2,4-dimethyl-	000105-67-9	15.5515	0.0000	1.9643(96)	2.962(89)	3.1428(96)	2.8762(96)
Benzene, (1-methyl-2-cyclopropen-1-yl)-	065051-83-4	15.6998	0.0000	0.0000	1.6594(96)	0.0000	0.0000
1H-Indene, 1-methyl-	000767-59-9	15.7059	1.7441(97)	0.0000	0.0000	1.5446(96)	0.0000
2-Hydroxy-3-methylbenzaldehyde	000824-42-0	15.7118	0.0000	0.0000	0.0000	0.0000	1.2012(78)
3-Hydroxy-4-methylbenzaldehyde	057295-30-4	15.7176	0.0000	0.6792(91)	0.0000	0.0000	0.0000

Tar Species	CAS	RT (min)	Waste Tires	Beech Sawdust	W75B25	W50B50	W25B75
Cycloprop[a]indene, 1,1a,6,6a-tetrahydro-	015677-15-3	15.9492	0.0000	0.0000	0.0000	0.5717(95)	0.0000
2,3-Dihydroxybenzaldehyde	024677-78-9	15.955	0.0000	0.5757(90)	0.0000	0.0000	0.0000
1,3-Cycloheptadiene	004054-38-0	16.335	1.2939(60)	0.0000	0.0000	0.0000	0.0000
Naphthalene	000091-20-3	16.5071	0.9546(94)	0.0000	1.2046(93)	1.5008(94)	1.6198(86)
5-(Hydroxymethyl) dihydrofuran-2(3H)-one	010374-51-3	16.5663	0.0000	0.9895(83)	0.0000	0.0000	0.0000
3'-Methylacetophenone	000585-74-0	16.5782	1.3203(94)	0.0000	1.3851(94)	0.0000	0.0000
2'-Methylacetophenone	000577-16-2	16.5783	0.0000	0.0000	0.0000	1.3908(92)	1.5814(93)
Creosol	000093-51-6	16.8394	0.0000	0.732(96)	0.0000	0.0000	0.7061(95)
1,6-Dimethylhepta-1,3,5-triene	1000196-61-0	16.8572	0.0000	0.0000	0.3237(90)	0.4041(83)	0.0000
1H-Indene, 2,3-dihydro-1,3-dimethyl-	004175-53-5	16.9937	0.0000	0.0000	0.3846(95)	0.0000	0.0000
1H-Indene, 2,3-dihydro-1,6-dimethyl-	017059-48-2	16.9939	0.5666(93)	0.0000	0.0000	0.0000	0.0000
Catechol	000120-80-9	17.0413	0.0000	2.5739(94)	0.0000	1.4715(94)	1.8479(95)
1,4:3,6-Dianhydro-.alpha.-d-glucopyranose	1000098-14-8	17.2014	0.0000	1.3043(96)	0.0000	0.7097(93)	1.1119(94)
3,4-Anhydro-d-galactosan	1000129-96-9	17.5101	0.0000	0.4641(64)	0.0000	0.0000	0.0000
Benzothiazole	000095-16-9	17.6705	3.2225(95)	0.0000	2.0255(95)	1.5103(94)	1.4725(64)
2,3-Anhydro-d-mannosan	1000129-98-0	17.694	0.0000	0.9128(80)	0.0000	0.0000	0.0000
5-Hydroxymethylfurfural	000067-47-0	17.7713	0.0000	0.8255(96)	0.0000	0.0000	0.4993(96)
Phenol, 2-ethyl-6-methyl-	001687-64-5	18.1512	0.0000	0.0000	0.0000	0.878(91)	0.0000
Phenol, 2-(1-methylethyl)-	000088-69-7	18.157	0.0000	0.9823(50)	0.0000	0.0000	0.0000
Phenol, 3-(1-methylethyl)-	000618-45-1	18.1571	0.0000	0.0000	0.0000	0.0000	1.0875(78)
1H-Indene, 1,1-dimethyl-	018636-55-0	18.3054	1.4044(92)	0.0000	1.4348(93)	0.0000	0.0000
1H-Indene, 1,3-dimethyl-	002177-48-2	18.5726	0.7978(96)	0.0000	0.8077(96)	0.8782(95)	1.4767(96)
1,2-Benzenediol, 3-methoxy-	000934-00-9	18.7327	0.0000	0.8007(97)	0.0000	0.0000	0.0000
1H-Indene, 4,7-dimethyl-	006974-97-6	18.7387	0.0000	0.0000	1.2831(93)	0.0000	0.0000
1H-Cyclopropa[b]naphthalene, 1a,2,7,7a-tetrahydro-	006571-72-8	18.7388	0.0000	0.0000	0.0000	1.1985(89)	0.0000
1H-Indene, 2,3-dimethyl-	004773-82-4	18.7389	1.4815(91)	0.0000	0.0000	0.0000	0.0000
Naphthalene, 1,2-dihydro-3-methyl-	002717-44-4	18.8753	0.0000	0.0000	0.0000	0.8551(78)	0.0000
1,4-Benzenedicarboxaldehyde, 2-methyl-	027587-17-3	18.8753	0.0000	0.0000	0.0000	0.0000	0.569(94)
Hydroquinone	000123-31-9	19.1007	0.0000	0.3634(92)	0.0000	0.0000	0.437(90)
o-Tolylacetic acid	000644-36-0	19.2313	0.0000	0.0000	0.5664(52)	0.0000	0.0000
1H-Inden-1-one, 2,3-dihydro-	000083-33-0	19.2314	0.0000	0.0000	0.0000	0.0000	0.6358(70)
Benzoic acid, 2,6-dimethyl-	000632-46-2	19.2374	0.0000	0.0000	0.0000	0.7595(50)	0.0000
4-Isopropylthiophenol	004946-14-9	19.2788	0.0000	0.7332(81)	0.0000	0.0000	0.0000

Tar Species	CAS	RT (min)	Waste Tires	Beech Sawdust	W75B25	W50B50	W25B75
1,2-Benzenediol, 4-methyl-	000452-86-8	19.5874	0.0000	0.7049(95)	0.0000	0.0000	0.4079(91)
Naphthalene, 1-methyl-	000090-12-0	19.6468	2.8031(96)	0.0000	2.7221(96)	2.5041(96)	0.0000
Naphthalene, 2-methyl-	000091-57-6	19.6469	0.0000	0.0000	0.0000	0.0000	1.2358(96)
Benzaldehyde, 2-hydroxy-3-methoxy-	000148-53-8	19.9732	0.0000	0.6507(96)	0.0000	0.0000	0.0000
Phthalic anhydride	000085-44-9	20.1394	0.0000	0.5245(64)	0.0000	0.0000	0.0000
2-Methoxy-4-vinylphenol	007786-61-0	20.2166	0.0000	1.2301(96)	0.0000	0.0000	0.0000
(1-Methylpenta-1,3-dienyl)benzene	116669-49-9	20.3651	0.0000	0.0000	0.0000	0.3758(60)	0.0000
3-Methoxy-5-methylphenol	003209-13-0	20.3946	0.0000	0.3293(94)	0.0000	0.0000	0.0000
Piperonal	000120-57-0	20.6024	0.0000	0.3817(72)	0.0000	0.0000	0.0000
Benzoic acid, 4-formyl-	000619-66-9	20.6084	0.0000	0.0000	0.0000	0.0000	0.622(60)
1,4-Benzenediol, 2-methyl-	000095-71-6	20.9822	0.0000	0.5712(86)	0.0000	0.0000	0.0000
Orcinol	000504-15-4	20.9824	0.0000	0.0000	0.0000	0.0000	0.4548(64)
Phenol, 2,6-dimethoxy-	000091-10-1	21.1959	0.0000	2.9332(97)	0.0000	0.4399(96)	1.6083(97)
Formic acid, 2,6-dimethoxyphenyl ester	1000368-91-0	21.4096	0.0000	0.4142(58)	0.0000	0.0000	0.0000
Phenol, 2-(1,1-dimethylethyl)-5-methyl-	000088-60-8	21.4511	0.0000	0.0000	0.4471(74)	0.0000	0.0000
Benzene, 1-butyl-4-methoxy-	018272-84-9	21.4573	0.4964(80)	0.0000	0.0000	0.0000	0.0000
1,2,3-Trimethylindene	004773-83-5	21.8193	0.7826(94)	0.0000	0.7991(95)	0.675(94)	0.0000
2-Methoxy-5-methylphenol	001195-09-1	22.0565	0.0000	0.309(83)	0.0000	0.0000	0.0000
Benzo[b]thiophene, 2,7-dimethyl-	016587-40-9	22.1456	0.0000	0.0000	0.8591(90)	0.0000	0.0000
Benzo[b]thiophene, 2,5-dimethyl-	016587-48-7	22.1457	0.0000	0.0000	0.0000	0.0000	0.5633(93)
Benzo[b]thiophene, 3,5-dimethyl-	001964-45-0	22.1458	0.7438(87)	0.0000	0.0000	0.8183(87)	0.0000
Benzaldehyde, 3-hydroxy-4-methoxy-	000621-59-0	22.3949	0.0000	1.54(97)	0.0000	0.0000	0.0000
Vanillin	000121-33-5	22.395	0.0000	0.0000	0.0000	0.0000	1.4708(96)
Naphthalene, 2,7-dimethyl-	000582-16-1	22.5729	0.0000	0.0000	1.9197(93)	0.0000	0.8792(89)
Naphthalene, 1,4-dimethyl-	000571-58-4	22.5791	1.352(93)	0.0000	1.3553(97)	0.0000	0.0000
4,7-Methanoisobenzofuran-1,3-dione, 3a,4,7,7a-tetrahydro, 5-methyl-	000117-40-8	22.7036	0.0000	0.0000	0.0000	0.0000	2.4292(90)
4,7-Methanoisobenzofuran-1,3-dione, 3a,4,7,7a-tetrahydro-	000826-62-0	22.8579	0.0000	0.0000	0.0000	0.0000	0.351(81)
Naphthalene, 1,3-dimethyl-	000575-41-7	22.941	0.0000	0.0000	0.0000	1.6042(95)	0.0000
Naphthalene, 2,3-dimethyl-	000581-40-8	22.9471	0.4317(97)	0.0000	0.0000	0.0000	0.0000
Naphthalene, 1,6-dimethyl-	000575-43-9	23.0241	0.0000	0.0000	0.0000	0.0000	0.8413(96)
Naphthalene, 2,6-dimethyl-	000581-42-0	23.0301	1.335(97)	0.0000	0.0000	1.196(98)	0.0000
2,3-Trimethylene-4-pyrone	1000427-87-0	23.208	0.0000	0.4965(53)	0.0000	0.0000	0.0000
Quinoline, 2,4-dimethyl-	001198-37-4	23.5166	0.0000	0.0000	0.6748(87)	0.476(81)	0.0000

Tar Species	CAS	RT (min)	Waste Tires	Beech Sawdust	W75B25	W50B50	W25B75
Quinoline, 2,7-dimethyl-	000093-37-8	23.5168	0.6998(90)	0.0000	0.0000	0.0000	0.0000
Biphenylene	000259-79-0	23.6591	0.0000	0.0000	0.3117(50)	0.0000	0.0000
3,5-Dimethoxy-4-hydroxytoluene	006638-05-7	23.6592	0.0000	1.1876(97)	0.0000	0.0000	0.5515(95)
Phenol, 2-methoxy-4-(1-propenyl)-	000097-54-1	23.7422	0.0000	0.6017(98)	0.0000	0.0000	0.0000
D-Allose	002595-97-3	24.4901	0.0000	14.163(90)	0.0000	4.8779(64)	4.0768(64)
Apocynin	000498-02-2	24.5969	0.0000	0.6498(93)	0.0000	0.0000	0.0000
Durohydroquinone	000527-18-4	24.597	0.0000	0.0000	0.0000	0.0000	0.4137(53)
Naphthalene, 1-(1-methylethyl)-	006158-45-8	24.864	0.0000	0.0000	0.3477(53)	0.0000	0.0000
Naphthalene, 1,4,5-trimethyl-	002131-41-1	25.06	0.3385(55)	0.0000	0.0000	0.0000	0.0000
2(3H)-Naphthalenone,4,4a,5,6,7,8-hexahydro-4a-methyl-	000826-56-2	25.2974	0.0000	0.0000	0.0000	0.0000	0.5317(53)
4(5H)-Benzofuranone, 6,7-dihydro-3,6-dimethyl-, (R)-	000529-63-5	25.3032	0.0000	0.7195(70)	0.0000	0.0000	0.0000
2,5-Dihydroxy-4-methoxyacetophenone	1000422-88-0	25.6178	0.0000	0.4453(72)	0.0000	0.0000	0.0000
Naphthalene, 1,6,7-trimethyl-	002245-38-7	25.6892	1.6187(98)	0.0000	1.5699(98)	1.27(98)	0.8569(98)
2-Propanone, 1-(4-hydroxy-3-methoxyphenyl)-	002503-46-0	25.6949	0.0000	0.3473(93)	0.0000	0.0000	0.0000
Phenol, 4-ethenyl-2,6-dimethoxy-	028343-22-8	26.514	0.0000	0.8224(96)	0.0000	0.0000	0.0000
Benzo[b]thiophene, 2-ethyl-5,7-dimethyl-	018428-05-2	26.9948	0.3301(70)	0.0000	0.3946(89)	0.0000	0.0000
2,2'-Ethylidenebis(5-methylfuran)	003209-79-8	26.9949	0.0000	0.0000	0.0000	0.4436(64)	0.0000
Benzene, 1-ethyl-3,5-diisopropyl-	015181-13-2	26.9949	0.0000	0.0000	0.0000	0.0000	0.475(50)
2-(Methylmercapto)benzothiazole	000615-22-5	27.2798	0.0000	0.0000	1.7408(99)	1.4546(99)	0.9908(99)
Phenol, 2,6-dimethoxy-4-(2-propenyl)-	006627-88-9	27.4103	0.0000	0.3507(95)	0.0000	0.0000	0.0000
(E)-2,6-Dimethoxy-4-(prop-1-en-1-yl)phenol	020675-95-0	28.5024	0.0000	1.4515(98)	0.0000	0.0000	0.0000
Benzaldehyde, 4-hydroxy-3,5-dimethoxy-	000134-96-3	28.627	0.0000	1.4901(98)	0.0000	0.0000	0.8434(95)
2(3H)-Benzothiazolone	000934-34-9	28.6389	0.0000	0.0000	0.4826(95)	0.7938(70)	0.0000
1H-Imidazole-4,5-dicarbonitrile, 2-phenyl-	1000338-11-6	29.5887	0.0000	0.0000	0.0000	0.3798(56)	0.0000
Ethanone, 1-(4-hydroxy-3,5-dimethoxyphenyl)-	002478-38-8	30.2474	0.0000	0.746(96)	0.0000	0.0000	0.0000
Syringylacetone	019037-58-2	31.0664	0.0000	0.7066(96)	0.0000	0.0000	0.0000
Anthracene	000120-12-7	31.1673	0.0000	0.0000	1.1104(95)	0.0000	0.0000
Phenanthrene	000085-01-8	31.1675	1.3049(96)	0.0000	0.0000	0.735(96)	0.0000
9H-Fluorene, 9-methylene-	004425-82-5	31.1675	0.0000	0.0000	0.0000	0.0000	0.6089(96)
n-Hexadecanoic acid	000057-10-3	34.889	0.0000	0.0000	0.0000	0.0000	1.068(99)
3,5-Dimethoxy-4-hydroxycinnamaldehyde	087345-53-7	35.2331	0.0000	0.2936(98)	0.0000	0.0000	0.0000
Fluoranthene	000206-44-0	36.6516	1.0629(98)	0.0000	1.0077(98)	0.7949(98)	0.5338(97)
Pyrene	000129-00-0	37.5716	0.8558(98)	0.0000	0.7741(98)	0.673(98)	0.4305(98)

Tar Species	CAS	RT (min)	Waste Tires	Beech Sawdust	W75B25	W50B50	W25B75
Phenol, 2,2'-methylenebis[6-(1,1-dimethylethyl)-4-methyl-	000119-47-1	41.5246	0.0000	0.0000	0.8101(99)	0.5467(99)	0.3778(99)
Chrysene	000218-01-9	41.9461	0.0000	0.0000	0.4197(98)	0.3994(98)	0.0000
Triphenylene	000217-59-4	41.9521	0.5027(98)	0.0000	0.0000	0.0000	0.0000

Table S2. Chromatographic concentration of detected organics (mol/ 10g samples) in gas produced from the pyrolysis of Waste tires and Beech sawdust.

Gaseous Species	MW (g/mol)	RT (min)	Waste tires			Beech sawdust		
			1	2	3	1	2	3
H_2	2.0	2.737	0.0113	0.0121	0.0117	0.0152	0.0148	0.0150
CH_4	16.0	9.813	0.0060	0.0065	0.0062	0.0073	0.0084	0.0079
CO	28.0	14.79	0.0052	0.0064	0.0064	0.0383	0.0364	0.0377

Table S3. Chromatographic concentration of detected organics (mol/ 10g samples) in gas produced from the pyrolysis of the Blends.

Gaseous Species	MW (g/mol)	RT (min)	W75B25			W50B50			W25B75		
			1	2	3	1	2	3	1	2	3
H_2	2.0	2.737	0.0121	0.0125	0.0120	0.0126	0.0117	0.0121	0.0126	0.0118	0.0122
CH_4	16.0	9.813	0.0066	0.0075	0.0072	0.0079	0.0089	0.0074	0.0088	0.0073	0.0072
CO	28.0	14.79	0.0170	0.0159	0.0159	0.0255	0.0214	0.0262	0.0326	0.0336	0.0327
